# Trends and disparities in hepatitis B burden in the Western Pacific Region, 2015–2024: a subregional analysis using modelled data

**DOI:** 10.1016/j.lanwpc.2026.101991

**Published:** 2026-09-24

**Authors:** Hsu Thinzar Maung, Mohamed Amine Ghrabi, Fukushi Morishita, Rajendra Prasad Hubraj Yadav, Huong Thi Giang Tran, Kiyohiko Izumi

**Affiliations:** a World Health Organization Regional Office for the Western Pacific, Manila, Philippines; b School of Tropical Medicine and Global Health, Nagasaki University, Nagasaki, Japan

**Keywords:** Hepatitis B, Western Pacific Region, Disease burden, Elimination, Vaccination, Mortality

## Abstract

**Background:**

The Western Pacific Region (WPR) carries one of the world's largest hepatitis B virus (HBV) burdens, but elimination progress remains uneven. We assessed changes in HBV burden and heterogeneity in elimination progress across the WPR during 2015–2024.

**Methods:**

We conducted a regional ecological study using standardised modelled estimates from the Center for Disease Analysis Foundation for 34 WPR countries and areas from 2015 to 2024. Country-specific temporal trends were quantified using log-linear regression to estimated annual percentage change (EAPC) with 95% CIs. We assessed incidence–mortality divergence, differences in EAPCs across analytical subregions, associations between vaccination coverage and HBV burden, and sensitivity to influential observations, shared model trajectories, non-linearity, and alternative country classification.

**Findings:**

Regional HBV prevalence declined from 5.28% (95% UI 4.23–6.34) in 2015 to 4.58% (3.66–5.49) in 2024, and chronic HBV incidence declined from 6.32 to 3.16 per 100,000 population. Under-five prevalence plateaued at approximately 0.28–0.29% from 2019, remaining above the WHO 2030 target of ≤0.1%. Crude modelled HBV mortality increased from 22.88 to 24.70 per 100,000. Of 34 countries and areas, 55.9% had declining chronic incidence alongside increasing mortality. Incidence and mortality EAPCs were modestly correlated (r = 0.39, 95% CI 0.06–0.64; p = 0.023), while evidence of systematic differences across analytical subregions was limited.

**Interpretation:**

Declining HBV incidence has not been accompanied by declining mortality in many WPR countries and areas. Elimination monitoring should therefore assess transmission and mortality as distinct outcomes and account for substantial country-level heterogeneity.

**Funding:**

World Health Organization.

**Research in context**

**Evidence before this study**

We searched PubMed and reviewed WHO reports, Polaris Observatory modelling studies, regional analyses, and relevant reference lists for evidence on hepatitis B virus (HBV) burden and elimination progress in the Western Pacific Region (WPR) from October 2024 to June 2026, using combinations of the terms “hepatitis B”, “Western Pacific Region”, “vaccination”, “mortality”, “incidence”, “disease burden”, and “elimination”. Searches were supplemented by relevant WHO and Center for Disease Analysis Foundation (CDA Foundation) publications identified through review of global hepatitis reporting. Existing evidence shows that the WPR carries a substantial proportion of the global HBV burden and has achieved major reductions in childhood infection following expansion of hepatitis B vaccination. Recent WHO assessments have documented declining HBV incidence alongside insufficient progress in reducing HBV-related mortality, as well as persistent gaps in diagnosis and treatment. However, regional aggregates can obscure substantial differences between countries and areas. We identified limited analyses that systematically quantified country-specific temporal trends in chronic HBV incidence, HBV-related mortality, hepatocellular carcinoma, and decompensated cirrhosis across the WPR and formally examined whether declining incidence has occurred alongside increasing mortality. Comparative assessments of these trajectories across WPR settings and against WHO elimination milestones also remain limited.

**Added value of this study**

This ecological study extends previous WHO and Polaris Observatory reporting of modelled HBV estimates by applying a common analytical framework to 34 WPR countries and areas over 2015–2024. Rather than relying only on changes between individual years or regional aggregates, we quantified country-specific temporal trends in chronic HBV incidence, HBV-related mortality, hepatocellular carcinoma, and decompensated cirrhosis using estimated annual percentage changes and their 95% CIs. We then formally characterised incidence–mortality trajectories and examined their association, finding that 19 (55.9%) of 34 countries and areas were classified as having declining chronic HBV incidence alongside increasing mortality based on the prespecified EAPC criteria. We additionally tested differences in temporal trends across analytical subregions, examined ecological associations between vaccination coverage and changes in HBV burden, benchmarked country-level progress against WHO elimination milestones and targets, and assessed robustness to influential observations, shared model-derived trajectories, departures from log-linearity, and an alternative income-based country classification. This approach identifies heterogeneity and divergence in HBV trajectories that are not apparent from WPR-wide estimates alone.

**Implications of all the available evidence**

Taken together, available evidence indicates substantial progress in reducing HBV transmission in the WPR, but reductions in chronic HBV incidence have not been accompanied by contemporaneous reductions in HBV-related mortality in many countries and areas. This divergence should not be interpreted as evidence that declining incidence causes changes in mortality, nor can the present ecological analysis determine whether increasing mortality reflects population ageing, the accumulated burden of longstanding chronic infection, gaps in diagnosis and treatment, changes in ascertainment, or other factors. Instead, the findings show that prevention and mortality trajectories need to be monitored separately when assessing elimination progress. Sustained prevention, including timely birth-dose and routine infant vaccination, remains essential, while existing evidence also supports strengthening diagnosis, linkage to care, antiviral treatment, and long-term management of people already living with chronic HBV. Elimination strategies should therefore address both prevention of new infections and morbidity and mortality among people with existing chronic infection.

## Introduction

Viral hepatitis remains a major global public health challenge despite the availability of effective vaccines, diagnostics, and antiviral treatment.^1^ The 2026 World Health Organization (WHO) Global Hepatitis Report estimated that approximately 240 million people were living with chronic hepatitis B virus (HBV) infection in 2024 and that HBV caused about 1.1 million deaths from cirrhosis and liver cancer, representing a 17% increase in HBV-related mortality since 2015.^1^ Although new HBV infections have declined globally, progress remains insufficient to achieve elimination as a public health threat by 2030. WHO targets include a 90% reduction in new infections and a 65% reduction in hepatitis-related mortality compared with 2015.^1^^,^^2^

Chronic HBV infection can progress over decades to decompensated cirrhosis (DC) and hepatocellular carcinoma (HCC), with HCC accounting for approximately 80% of primary liver cancers globally.^1^^,^^3^^,^^4^ Universal infant immunisation, including a timely birth dose (HepB-BD) followed by a complete three-dose schedule (HepB3), remains central to preventing new HBV infections.^1^^,^^3^^,^^5^ These interventions have substantially reduced childhood infection, but reductions in transmission may not be accompanied by contemporaneous reductions in mortality because cirrhosis and HCC develop over many years among people with longstanding chronic infection. Incidence and mortality therefore represent related but temporally distinct dimensions of elimination progress.^1^^,^^2^

This distinction is particularly relevant to the Western Pacific Region (WPR), where an estimated 102 million people were living with HBV in 2024. Several WHO-priority countries contributing disproportionately to the global HBV burden or mortality, including China, the Philippines, Viet Nam and Indonesia, are also located within the region.^1^ The WPR encompasses diverse geographic, economic, and health-system contexts and thus, regional averages can obscure differences in elimination progress between countries and areas.^6^ Although vaccination coverage has remained comparatively high regionally, both coverage and HBV burden vary considerably between settings and disruption in HepB-BD coverage during the COVID-19 pandemic further illustrated the vulnerability of prevention programmes.^1^^,^^7^ The 2026 WHO Global Hepatitis Report provides updated global and regional estimates through 2024, but regional summaries do not establish whether divergent incidence and mortality trajectories occur consistently across individual WPR countries or whether countries have progressed similarly towards elimination. Country-level analysis is therefore needed to characterise this heterogeneity and progress towards WHO elimination targets.

Assessing these differences is complicated by limitations in empirical HBV surveillance because chronic infection is often asymptomatic and routine surveillance data remain limited in many settings.^8^^,^^9^ Modelled estimates, therefore, provide an important basis for tracking long-term trends and supporting elimination planning where directly observed data are incomplete. The GBD 2019 analysis estimated a 76.8% reduction in under-five HBV prevalence between 1990 and 2019,^8^ while the Polaris Observatory, part of the Center for Disease Analysis Foundation (CDA Foundation), has generated annual country-level estimates of HBV burden and intervention outcomes that have informed global and national assessments, including WHO Global Hepatitis Reports.^1^^,^^10^, ^11^, ^12^ However, modelled estimates depend on the availability and quality of country-specific inputs, and in data-limited settings, estimates may rely more heavily on regional comparators and external epidemiological assumptions. Therefore, they should be interpreted alongside local surveillance and programme context.

We conducted an ecological analysis of standardised CDA Foundation estimates for 34 WPR countries and areas during 2015–2024. We quantified country-specific temporal trends in chronic HBV incidence, HBV-related mortality, HCC, and DC incidence using estimated annual percentage changes; assessed whether declining incidence was accompanied by declining mortality across countries; compared trajectories across four analytical subregions; explored associations between vaccination coverage and changes in HBV burden; and assessed country-level progress against WHO 2025 interim milestones and 2030 targets. Sensitivity analyses examined the robustness of the findings to influential observations, shared model-derived trajectories, departures from log-linearity, and an alternative income-based country classification.

## Methods

### Study design and data sources

This ecological study assessed country-level modelled estimates of HBV burden and elimination progress across 34 countries and areas in the WPR from 2015 to 2024. Reporting followed the Guidelines for Accurate and Transparent Health Estimates Reporting (GATHER).^13^

Epidemiological estimates were obtained from the CDA Foundation, which uses the progress dynamic disease-progression model within the Polaris Observatory to generate country-specific estimates of HBV epidemiology and clinical outcomes. The model integrates data from published literature, national reports, WHO and UNICEF immunisation datasets, and input from national experts. The modelling framework, calibration, and data inputs have been described previously.^10^^,^^11^^,^^14^

The availability and quality of empirical input data vary between countries and areas. Where country-specific data are limited, the progress modelling framework may use regional comparator estimates and external epidemiological assumptions to generate country-level estimates, resulting in identical or near-identical model-derived values or trajectories for some settings. Such estimates should not be interpreted as independently observed country-level measurements. Countries and areas for which the CDA Foundation did not generate HBV estimates because of insufficient data were excluded. Indonesia was not included because data extraction and modelling for this analysis were completed before its reassignment from the WHO South-East Asia Region to the WPR in May 2025.

### Subregion grouping

To examine heterogeneity within the WPR, the 34 countries and areas were grouped into four analytical subregions based on geographic proximity and commonly recognised regional distinctions. These study-specific groupings do not represent formal WHO subregional classifications and were intended to facilitate structured comparison across the heterogeneous WPR rather than imply epidemiology, socioeconomic conditions, health-system capacity, or programme homogeneity within groups.

The analytical subregions were:

• WPR-East Asia (WEA): China; the Republic of Korea; Japan; Mongolia.
• WPR-Southeast Asia (WSEA): Singapore; Brunei Darussalam; Malaysia; Cambodia; Lao People's Democratic Republic (Lao PDR); Viet Nam; Philippines.
• Australasia: Australia; New Zealand.
• Pacific Island Countries and Territories (PICTs): American Samoa; Guam; Northern Mariana Islands; New Caledonia; Nauru; Palau; French Polynesia; Fiji; Marshall Islands; Tonga; Tuvalu; Kiribati; Micronesia, Federated States of; Papua New Guinea (PNG); Solomon Islands; Samoa; Vanuatu; Cook Islands; Niue; Tokelau; Wallis and Futuna Islands.

In this study, the WPR refers to these 34 countries and areas, modelled by the CDA Foundation. Several member states were excluded due to data limitations; thus, findings require cautious interpretation.

### Outcomes and elimination targets

Epidemiological outcomes were total HBV prevalence (%), under-five HBV prevalence (%), chronic HBV incidence (per 100,000 population), HBV-related mortality (per 100,000 population), HBV-related HCC incidence (per 100,000 population), and HBV-related DC incidence (per 100,000 population). Mortality rates were not age-standardised and are reported as crude modelled estimates because age-standardised estimates were not available in the CDA Foundation dataset used for this analysis. Prevention indicators included HepB-BD vaccination coverage and HepB3 vaccination coverage.

Progress towards HBV elimination was assessed against the WHO 2025 interim milestones and 2030 targets for under-five HBV prevalence, HBV-related mortality, HepB-BD, and HepB3 coverage (Table 1). For countries implementing targeted rather than universal HepB-BD vaccination, birth-dose coverage was not compared directly with the universal coverage targets. Indicators for which data were unavailable were treated as missing rather than interpreted as failure to meet a target.

**Table 1:** WHO baseline values, interim milestones, and elimination targets used to benchmark HBV impact and programmatic indicators.

Key indicators	2020	2025	2030
Impact indicator			
Under-five HBV prevalence (%)	0.94	0.5	0.1
HBV-related mortality rate (per 100,000)	10	7	4
Programmatic Indicators			
HBV birth dose (HepB-BD) vaccination coverage (%)	50	70	90
HBV 3-dose (HepB3) vaccination coverage (%)	90	90	90

Adapted from the WHO Global Health Sector Strategy on Viral Hepatitis, 2016, and the WHO Interim guidance for country validation of HBV elimination 2021.^2^^,^^15^

### Statistical analyses

Regional and country-level trends during 2015–2024 were summarised descriptively. Regional estimates were derived directly from the WPR aggregate provided by the CDA Foundation. Country-level and subregional analyses included the 34 countries and areas with available data. Percentage changes in prevalence were calculated relative to 2015 values. Uncertainty intervals (UIs) for underlying modelled estimates were reported where provided by the CDA Foundation. UIs were available for prevalence estimates but not for the incidence, mortality, HCC, or DC estimates. These outcomes were therefore analysed as modelled point estimates. Population denominators used for rates per 100,000 population were obtained from the CDA Foundation analytical dataset and were applied consistently across countries and years.

Country-specific temporal trends in chronic HBV incidence, HBV-related mortality, HCC incidence, and DC incidence were quantified using log-linear regression. Calendar year was entered as the independent variable, and the natural logarithm of the annual rate as the dependent variable. The log-linear approach summarised average relative change across ten annual observations and enabled comparison between countries with different baseline rates. The estimated annual percentage change (EAPC) was calculated as 100\times[\exp(\beta)-1], where \beta was the regression coefficient for calendar year. Corresponding 95% CIs were derived from the regression coefficient using the t-distribution. Residual serial correlation in the country-specific log-linear models for chronic HBV incidence and HBV-related mortality was assessed using lag-1 residual autocorrelation and the Durbin–Watson test. Given the limited number of annual observations per country (n = 10), these diagnostics were used to assess potential temporal dependence rather than to establish residual independence.

The EAPC represents the estimated average annual percentage change over 2015–2024, rather than a constant annual change. The 95% CIs quantify uncertainty around the fitted temporal trend conditional on the annual modelled point estimates and do not incorporate uncertainty in the underlying CDA Foundation estimates. Because annual estimates were generated within the same dynamic modelling framework, temporal dependence and model smoothing may result in greater apparent precision of the fitted EAPCs than would be supported by independently observed annual epidemiological data. A declining trend was defined as an EAPC with a 95% CI entirely below zero, an increasing trend as an EAPC with a 95% CI entirely above zero, and no clear trend when the 95% CI included zero. HCC and DC EAPCs could not be estimated for countries or areas with rates of zero throughout 2015–2024 because the rates could not be log-transformed.

To assess the relationship between chronic HBV incidence and HBV-related mortality trends, country-level EAPCs were cross-classified according to these trend categories. A discordant incidence–mortality trajectory was defined a priori as declining chronic HBV incidence alongside increasing HBV-related mortality. The proportion of countries and areas with this trajectory was calculated overall and within each analytical subregion. Fisher's exact test was used to assess differences in the frequency of this trajectory across subregions because of the small and unequal numbers of countries and areas within groups.

The association between country-level chronic HBV incidence and HBV-related mortality EAPCs was further assessed using Pearson correlation and simple linear regression, with mortality EAPC as the dependent variable and incidence EAPC as the independent variable. The regression coefficient was interpreted as the difference in mortality EAPC associated with a one percentage-point difference in chronic HBV incidence EAPC, and R^2^ was used to quantify the proportion of between-country variation in mortality EAPC explained by incidence EAPC.

Differences in country-level EAPC distributions across WEA, WSEA, Australasia, and PICTs were assessed using the Kruskal–Wallis test due to small and unequal subregional sample sizes. Where an omnibus test indicated evidence of differences across groups, Dunn's pairwise comparisons with Holm adjustment for multiple testing were performed.

Exploratory analyses assessed associations between hepatitis B vaccination coverage and changes in modelled HBV burden during 2015–2024. Analyses were restricted to countries and areas with HepB-BD and HepB3 coverage in both 2015 and 2024. Change in vaccination coverage was calculated as the absolute percentage-point difference between 2015 and 2024. Associations of baseline vaccination coverage in 2015 and changes in vaccination coverage with percentage change in under-five HBV prevalence and chronic HBV incidence EAPC were assessed using Spearman rank correlation because vaccination coverage was bounded and exhibited substantial ceiling effects. Holm adjustment was applied across the eight exploratory comparisons to account for multiple testing. All tests were two-sided, and P < 0.05 was considered to indicate statistical significance.

### Sensitivity analyses

Several sensitivity analyses were undertaken to assess the robustness of the primary analyses. First, the incidence-mortality Pearson correlation and linear regression were repeated after excluding the Marshall Islands, which had the highest HBV-related mortality EAPC.

Second, because several PICTs had identical model-derived chronic HBV incidence and mortality trajectories, countries and areas sharing an identical pair of trajectories were represented by a single analytical observation, and the incidence–mortality correlation and regression were repeated.

Third, the suitability of the log-linear representation of temporal trends was assessed by comparing each country-specific log-linear model with one that included an additional quadratic term for calendar year. For this sensitivity analysis, substantial departure from log-linearity was defined a priori as both P < 0.05 for the quadratic term and an increase in R^2^ of greater than 0.05 compared with the corresponding log-linear model. The incidence–mortality correlation and regression were subsequently repeated after excluding countries and areas meeting these criteria for chronic HBV incidence.

For the exploratory association between change in HepB3 coverage and chronic HBV incidence EAPC, the analysis was repeated after excluding Japan because of its marked increase in HepB3 coverage during the study period. Additionally, to assess sensitivity to the analytical subregional classification, country-level EAPCs were compared using World Bank fiscal year 2025 (FY2025) income classifications, which are based on calendar-year 2023 gross national income (GNI) per capita. Countries and areas without an FY2025 World Bank income classification were excluded. Chronic HBV incidence, HBV-related mortality, HCC incidence, and DC incidence EAPCs were compared across lower-middle-income, upper-middle-income, and high-income groups using Kruskal–Wallis tests. Post-hoc pairwise comparisons were planned only when the omnibus test indicated evidence of differences across income groups.

For contextual comparison, selected regional estimates and epidemiological patterns were compared descriptively with the WHO Global hepatitis report 2026^1^ and an independent analysis of GBD 2021 estimates^16^ (Supplementary Table S6).

All statistical analyses were conducted using R version 4.6.0.

### Ethics statement

This study was a secondary analysis of aggregate, country-level modelled estimates and did not involve individual-level data, participant recruitment, or contact with human participants. Ethical approval and informed consent were therefore not required. Selected indicators are publicly available through the CDA Foundation; additional variables used in the analysis were accessed under a data-sharing agreement with the CDA Foundation.

### Role of the funding source

The funder had no role in study design, data collection, data analysis, data interpretation, or writing of the manuscript.

## Results

### HBV disease burden declined, while HBV-related mortality increased

Between 2015 and 2024, total HBV prevalence in the WPR declined from 5.28% (95% UI 4.23–6.34) to 4.58% (3.66–5.49), corresponding to 100.8 million–89.7 million people living with HBV, respectively. Under-five HBV prevalence declined from 0.40% (95% UI 0.32–0.48) in 2015 to 0.28% (0.23–0.34) in 2024 and plateaued after 2019 at approximately 0.28–0.29%. Chronic HBV incidence declined from 6.32 to 3.16 per 100,000 population in 2024. In contrast, HBV-related mortality increased from 22.88 to 24.70 per 100,000, corresponding to an increase from 436,363 to 484,055 estimated deaths (Table 2, Fig. 1).

**Table 2:** HBV prevalence, chronic HBV incidence, and liver-related mortality in the WPR (2015–2024).

Year	Population (n)	HBV total prevalence		HBV under-five prevalence		Chronic HBV incidence		Liver-related HBV mortality	
		Cases (n)	Rate (%) (95% UI)	Cases (n)	Rate (%) (95% UI)	Cases (n)	Rate per 100,000	Cases (n)	Rate per 100,000
2015	1,907,244,494	100,770,811	5.28 (4.23–6.34)	609,140	0.40 (0.32–0.48)	120,608	6.32	436,363	22.88
2016	1,919,819,236	99,646,994	5.19 (4.15–6.23)	545,475	0.35 (0.28–0.43)	100,510	5.24	444,938	23.18
2017	1,932,608 785	98,499,108	5.10 (4.08–6.12)	499,191	0.32 (0.26–0.39)	92,424	4.78	447,788	23.17
2018	1,943,423,734	97,327,168	5.01 (4.01–6.01)	477,634	0.31 (0.25–0.38)	99,945	5.14	452,906	23.31
2019	1,951,805,091	96,133,298	4.93 (3.94–5.91)	433,255	0.29 (0.23–0.35)	81,474	4.17	459,373	23.54
2020	1,957,665,212	94,912,360	4.85 (3.88–5.82)	406,290	0.29 (0.23–0.34)	73,858	3.77	466,735	23.84
2021	1,960,344,181	93,667,837	4.78 (3.82–5.73)	369,964	0.28 (0.22–0.33)	74,861	3.82	472,204	24.09
2022	1,961,191,878	92,359,867	4.71 (3.77–5.65)	347,745	0.28 (0.22–0.34)	62,481	3.19	476,686	24.31
2023	1,960,879,728	91,043,739	4.64 (3.71–5.57)	331,690	0.29 (0.23–0.35)	65,749	3.35	480,768	24.52
2024	1,959,798,056	89,681,695	4.58 (3.66–5.49)	298,624	0.28 (0.23–0.34)	61,958	3.16	484,055	24.70

Values are presented for all ages and for children younger than five years where applicable. For consistency, all values were rounded to two decimal places. Under-five HBV prevalence was estimated from the population aged younger than 5 years as the denominator; the Population (n) column represents the total regional population.

**Fig. 1:**
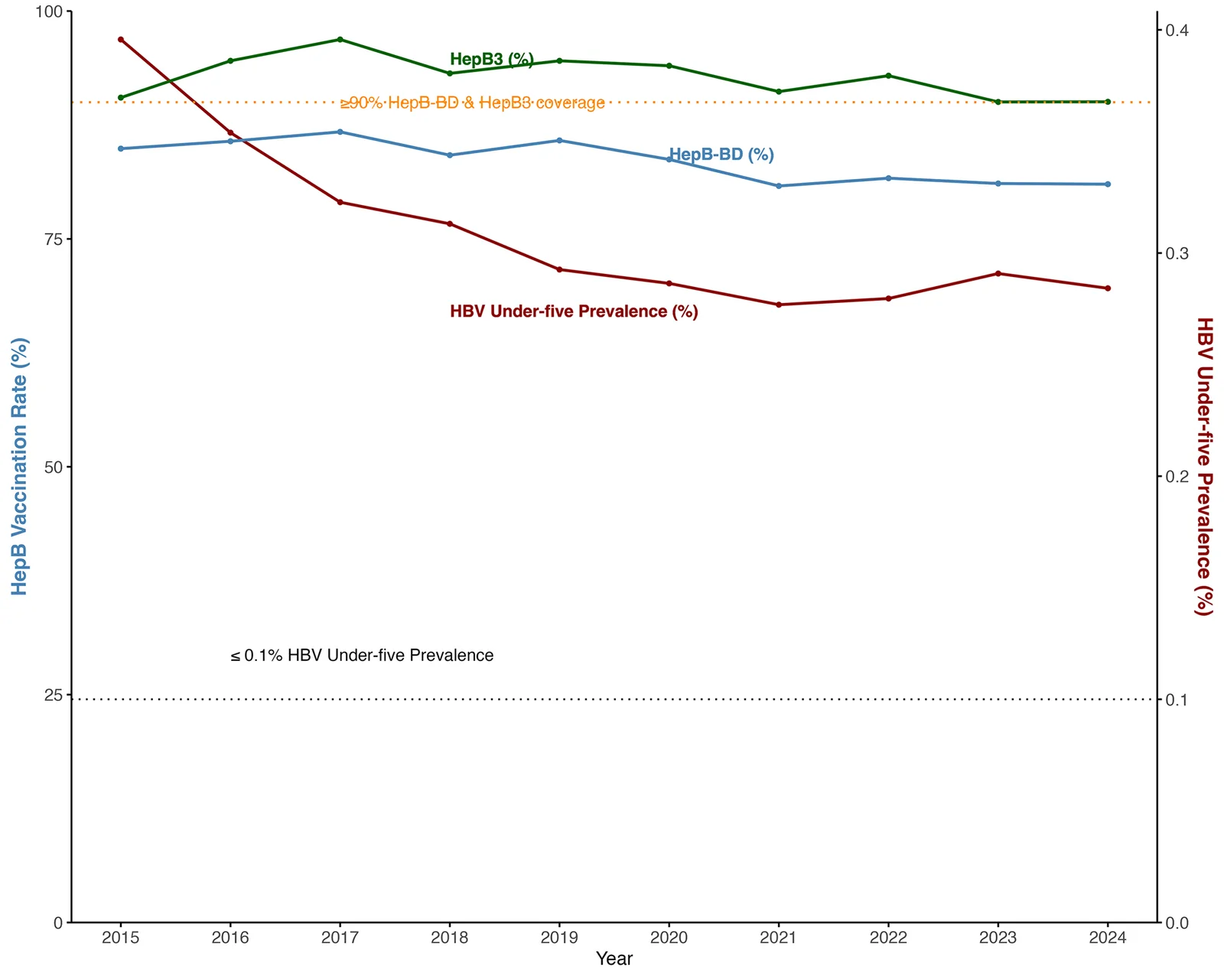
**Trends in hepatitis B vaccination coverage and under-five HBV prevalence in the WPR, 2015**–**2024.** Regional trends in hepatitis B birth-dose vaccination coverage (HepB-BD), hepatitis B three-dose vaccination coverage (HepB3), and under-five HBV prevalence in the Western Pacific Region from 2015 to 2024. Vaccination coverage is shown on the left y-axis, and under-five HBV prevalence on the right y-axis. Horizontal reference lines indicate the WHO 2030 targets: ≥90% coverage for HepB-BD and HepB3, and ≤0.1% under-five HBV prevalence. HBV = hepatitis B virus. HepB-BD = hepatitis B birth dose. HepB3 = hepatitis B three-dose vaccination coverage. WPR = Western Pacific Region.

Country-level changes in prevalence varied substantially within and across the four analytical subregions (Table 3). Under-five HBV prevalence declined in all WEA and WSEA countries and both countries in Australasia, with China (0.08%), the Republic of Korea (0.03%), Japan (0.01%), Australia (0.06%), and New Zealand (0.02%) reaching ≤0.1% by 2024. However, prevalence remained above 1% in Brunei Darussalam (1.30%) and Viet Nam (1.03%). Patterns among PICTs were more heterogeneous. In 2024, under-five prevalence remained above 1% in New Caledonia (2.17%), PNG (2.38%), Vanuatu (1.79%), Kiribati (1.71%), Solomon Islands (1.37%), Tuvalu (1.26%), Guam (1.17%), Northern Mariana Islands (1.17%), Nauru (1.17%), and Palau (1.17%), whereas French Polynesia had reached 0.03%. Under-five prevalence increased between 2015 and 2024 in PNG (+24.61%), New Caledonia (+19.89%), Samoa (+16.21%), and Vanuatu (+2.87%), while declining in the remaining PICTs. Total HBV prevalence declined in most countries and areas across the WPR, with increases estimated only in the Marshall Islands (+22.73%) and Tuvalu (+1.49%) (Table 3).

**Table 3:** Country-level HBV prevalence and vaccination coverage in the WPR (2015–2024).

Country	Total population prevalence rate (%, 95% UI)			Under-five prevalence rate (%, 95% UI)			Vaccination coverage in 2024 (%)	
	2015	2024	% Change	2015	2024	% Change	HepB-BD	HepB3
WEA								
China	5.73 (5.30–6.00)	4.98 (4.60–5.20)	−13.09	0.23 (0.12–0.32)	0.08 (0.04–0.12)	−63.22	92.95	97.10
Republic of Korea	2.94 (2.26–4.34)	2.66 (2.05–2.93)	−9.52	0.14 (0.10–0.20)	0.03 (0.02–0.05)	−78.57	92.00	97.00
Japan^a^	0.87 (0.75–0.88)	0.73 (0.63–0.73)	−16.09	0.05 (0.05–0.07)	0.01 (0.01–0.01)	−80	0.23	95.00
Mongolia	6.87 (6.36–7.27)	5.38 (4.98–5.69)	−21.69	0.52 (0.43–1.02)	0.35 (0.29–0.68)	−32.69	98.51	96.46
WSEA								
Singapore	2.76 (2.15–2.72)	2.33 (1.82–2.30)	−15.58	0.17 (0.13–0.22)	0.05 (0.04–0.07)	−70.59	91.40	96.80
Brunei Darussalam	5.74 (4.59–6.88)	4.99 (3.99–5.99)	−13.07	1.91 (1.53–2.29)	1.30 (1.04–1.57)	−31.94	95.70	99.93
Malaysia	2.82 (2.39–3.16)	2.18 (1.84–2.44)	−22.69	0.20 (0.15–0.29)	0.08 (0.06–0.12)	−60	99.00	99.00
Cambodia	3.15 (2.26–4.34)	2.70 (1.94–3.73)	−14.29	0.44 (0.32–0.72)	0.40 (0.29–0.66)	−9.09	85.00	82.00
Lao PDR	4.48 (3.20–5.10)	3.86 (2.80–4.40)	−13.84	1.74 (1.30–2.00)	0.59 (0.40–0.70)	−66.09	77.65	95.52
Viet Nam	7.27 (6.56–10.28)	6.17 (5.58–8.73)	−15.13	1.46 (1.16–2.24)	1.03 (0.82–1.58)	−29.45	81.82	64.90
Philippines	5.60 (2.71–9.90)	4.76 (2.30–8.42)	−15	0.99 (0.58–2.04)	0.84 (0.49–1.72)	−15.15	57.37	76.81
Australasia								
Australia	0.95 (0.50–1.58)	0.81 (0.43–1.34)	−14.74	0.09 (0.04–0.19)	0.06 (0.03–0.12)	−33.33	90.72	94.11
New Zealand^a^	2.10 (1.39–3.20)	1.59 (1.05–2.42)	−24.29	0.09 (0.06–0.14)	0.02 (0.01–0.03)	−77.78	0.71	88.29
PICTs								
American Samoa	3.23 (2.59–3.88)	2.88 (2.30–3.45)	−10.84	0.47 (0.37–0.56)	0.38 (0.31–0.46)	−19.15	–	–
Guam	6.66 (5.33–7.99)	5.94 (4.75–7.12)	−10.81	1.50 (1.20–1.80)	1.17 (0.94–1.40)	−22	–	–
Northern Mariana Islands	6.66 (5.33–7.99)	5.94 (4.75–7.12)	−10.81	1.50 (1.20–1.80)	1.17 (0.94–1.40)	−22	–	–
New Caledonia	4.82 (3.86–5.79)	4.11 (3.29–4.93)	−14.73	1.81 (1.44–2.17)	2.17 (1.73–2.60)	19.89	–	–
Nauru	6.66 (5.33–7.99)	5.94 (4.75–7.12)	−10.81	1.50 (1.20–1.80)	1.17 (0.94–1.40)	−22	99.00	97.98
Palau	6.66 (5.33–7.99)	5.94 (4.75–7.12)	−10.81	1.50 (1.20–1.80)	1.17 (0.94–1.40)	−22	99.12	97.51
French Polynesia	0.88 (0.70–1.06)	0.78 (0.62–0.93)	−11.36	0.05 (0.04–0.06)	0.03 (0.02–0.03)	−40	98.00	99.00
Fiji	1.97 (1.71–2.11)	1.67 (1.44–1.78)	−15.23	0.29 (0.23–0.41)	0.25 (0.20–0.35)	−13.79	39.12	95.24
Marshall Islands	4.18 (3.83–4.57)	5.13 (4.69–5.60)	22.73	1.20 (1.04–1.52)	0.93 (0.81–1.18)	−22.5	92.54	88.62
Tonga	9.91 (8.81–10.98)	9.16 (8.13–10.14)	−7.57	1.26 (1.26–2.10)	0.66 (0.66–1.10)	−47.61	99.20	99.17
Tuvalu	8.08 (7.90–10.80)	8.20 (8.10–10.90)	1.49	1.38 (1.38–3.21)	1.26 (1.26–2.94)	−8.70	99.65	94.14
Kiribati	9.44 (6.70–10.93)	7.65 (5.43–8.86)	−18.96	1.97 (1.41–2.82)	1.71 (1.22–2.44)	−13.20	94.16	90.00
Micronesia, Federated States of	4.77 (4.20–5.20)	4.16 (3.70–4.50)	−12.79	1.04 (1.56–1.56)	0.52 (0.50–0.60)	−50	78.10	85.13
PNG	4.63 (4.10–5.90)	3.97 (3.60–5.10)	−14.26	1.91 (1.70–2.50)	2.38 (2.20–3.10)	24.61	26.20	39.84
Solomon Islands	10.00 (6.54–12.45)	7.50 (4.91–9.33)	−25	2.08 (1.63–3.19)	1.37 (1.07–2.10)	−34.14	75.29	83.51
Samoa	2.69 (2.30–2.80)	2.36 (2.00–2.40)	−12.27	0.37 (0.30–0.50)	0.43 (0.30–0.50)	16.21	92.00	83.00
Vanuatu	8.70 (4.62–14.20)	6.78 (3.60–11.07)	−22.07	1.74 (0.93–2.90)	1.79 (0.96–2.99)	2.87	76.00	72.00
Cook Islands	3.23 (2.59–3.88)	2.88 (2.30–3.45)	−10.84	0.47 (0.37–0.56)	0.38 (0.31–0.46)	−19.15	98.99	69.00
Niue	3.23 (2.59–3.88)	2.88 (2.30–3.45)	−10.84	0.47 (0.37–0.56)	0.38 (0.31–0.46)	−19.15	93.00	99.00
Tokelau	3.23 (2.59–3.88)	2.88 (2.30–3.45)	−10.84	0.47 (0.37–0.56)	0.38 (0.31–0.46)	−19.15	–	–
Wallis and Futuna Islands	3.23 (2.59–3.88)	2.88 (2.30–3.45)	−10.84	0.47 (0.37–0.56)	0.38 (0.31–0.46)	−19.15	–	–

Percentage changes are calculated as the relative change in prevalence from 2015 to 2024, using 2015 as the baseline.

For consistency, all values were rounded to two decimal places. Values smaller than 0.01 are reported as <0.01. Countries without data reflect data unavailability or lack of modelled estimates, rather than the true absence of cases or services. Some identical or near-identical values across countries and areas reflect the use of regional comparator estimates within the CDA Foundation modelling framework, where country-specific empirical data were limited. These estimates should therefore be interpreted as modelled country-level values rather than independently observed measurements.

a Japan and New Zealand implement targeted birth dose strategies rather than universal HepB-BD vaccination, which is reflected in their lower reported birth-dose coverage.

### Country-level trends in chronic HBV incidence and severe liver outcomes

Country-specific EAPCs demonstrated substantial heterogeneity during 2015–2024 (Table 4). Chronic HBV incidence was classified as declining in 22 of 34 countries and areas, while 12 had no clear trend. Declining trends occurred in all WEA and Australasia countries; six of seven WSEA countries, with Viet Nam showing no clear trend, and ten of 21 PICTs, with 11 having no clear trend. The largest declines occurred in Japan (EAPC −19.24%, 95% CI −22.21 to −16.16) and the Republic of Korea (−15.66%, 95% CI −18.52 to −12.69). By contrast, chronic incidence showed little evidence of change in PNG (−0.16%, 95% CI −1.57 to 1.28), New Caledonia (−0.41%, 95% CI −1.69 to 0.90), and several other PICTs.

**Table 4:** Country-level trends in chronic HBV incidence, HBV-related complications, and HBV-related mortality in the WPR (2015 and 2024).

Country	Chronic HBV incidence per 100,000			HBV related mortality rate per 100,000			HBV related HCC incidence rate per 100,000			HBV related DC incidence rate per 100,000		
	2015	2024	EAPC (%, 95% CI)	2015	2024	EAPC (%, 95% CI)	2015	2024	EAPC (%, 95% CI)	2015	2024	EAPC (%, 95% CI)
WEA												
China	3.60	1.19	−12.89 (−14.74 to −10.99)	25.89	27.79	0.80 (0.70–0.91)	19.50	20.78	0.79 (0.55–1.02)	9.03	8.98	0.11 (−0.41 to 0.63)
Republic of Korea	1.33	0.30	−15.66 (−18.52 to −12.69)	12.28	13.28	0.90 (0.82–0.99)	9.42	10.61	1.33 (1.27–1.40)	4.12	4.15	0.06 (−0.09 to 0.20)
Japan	0.52	0.09	−19.24 (−22.21 to −16.16)	5.73	4.29	−3.09 (−3.63 to −2.55)	3.99	3.55	−1.07 (−1.45 to −0.69)	1.29	0.83	−3.59 (−5.59 to −1.55)
Mongolia	13.40	6.36	−6.64 (−8.40 to −4.84)	17.56	21.36	2.23 (2.12–2.33)	13.67	16.37	2.13 (1.96–2.29)	6.82	8.14	2.19 (1.83–2.55)
WSEA												
Singapore	1.93	0.79	−8.30 (−9.56 to −7.03)	14.95	17.93	2.21 (1.80–2.63)	10.93	12.67	1.82 (1.41–2.22)	5.77	6.91	2.16 (1.75–2.57)
Brunei Darussalam	33.74	23.18	−3.81 (−6.12 to −1.45)	17.64	20.31	1.58 (1.53–1.64)	13.03	14.92	1.50 (1.36–1.65)	6.63	7.78	1.84 (1.58–2.11)
Malaysia	3.06	0.94	−12.32 (−13.34 to −11.29)	11.52	12.17	0.62 (0.56–0.68)	8.35	8.59	0.33 (0.27–0.38)	4.60	4.83	0.56 (0.50–0.63)
Cambodia	12.15	8.63	−2.39 (−4.70 to −0.03)	5.12	7.26	3.97 (3.85–4.09)	4.08	5.61	3.58 (3.44–3.72)	1.81	2.73	4.68 (4.46–4.90)
Lao PDR	31.35	11.70	−10.56 (−12.95 to −8.11)	7.84	8.99	1.56 (1.45–1.68)	5.88	6.87	1.73 (1.70–1.76)	2.71	3.27	2.16 (2.13–2.19)
Viet Nam	62.93	19.45	−2.06 (−5.90 to 1.94)	26.06	29.99	1.58 (1.45–1.70)	19.14	21.81	1.48 (1.34–1.62)	10.04	11.70	1.75 (1.49–2.00)
Philippines	33.45	12.29	−7.05 (−11.56 to −2.30)	13.10	16.82	2.84 (2.78–2.89)	10.19	12.87	2.63 (2.57–2.68)	4.83	6.45	3.27 (3.20–3.34)
Australasia												
Australia	1.51	0.77	−7.23 (−8.18 to −6.27)	3.35	3.70	1.06 (0.98–1.13)	2.50	2.80	1.15 (0.93–1.36)	1.15	1.24	0.54 (0.02–1.06)
New Zealand	0.78	0.26	−11.37 (−12.20 to −10.52)	13.12	11.60	−1.37 (−1.51 to −1.24)	9.28	8.19	−1.42 (−1.61 to −1.23)	4.83	4.10	−1.93 (−2.16 to −1.70)
PICTs												
American Samoa	12.74	7.11	−6.83 (−10.71 to −2.78)	14.11	18.35	2.97 (2.89–3.05)	11.35	12.83	1.35 (1.26–1.43)	5.67	6.42	1.34 (1.26–1.43)
Guam	39.39	18.18	−6.92 (−15.11 to 2.05)	22.81	28.21	2.37 (2.23–2.50)	16.98	20.86	2.25 (2.08–2.42)	9.10	11.32	2.70 (2.15–3.26)
Northern Mariana Islands	39.39	18.18	−7.26 (−15.11 to 2.05)	22.81	28.21	2.37 (2.23–2.50)	17.47	20.33	1.67 (1.59–1.75)	9.71	11.29	1.67 (1.59–1.75)
New Caledonia	58.70	55.68	−0.41 (−1.69 to 0.90)	16.47	17.75	0.84 (0.80–0.87)	12.01	12.99	0.67 (0.44–0.91)	6.36	6.83	1.02 (0.60–1.45)
Nauru	39.39	18.18	−6.93 (−15.11 to 2.05)	22.81	28.21	2.37 (2.23–2.50)	18.26	16.74	−0.90 (−1.07 to −0.73)	9.13	8.37	−0.90 (−1.07 to −0.73)
Palau	39.39	18.18	−6.93 (−15.11 to 2.05)	22.81	28.21	2.37 (2.23–2.50)	16.88	22.61	2.88 (0.47–5.35)	11.26	11.30	0.05 (0.01–0.09)
French Polynesia	0.75	0.28	−10.69 (−11.36 to −10.01)	4.77	5.99	2.58 (2.35–2.81)	3.60	4.26	2.24 (1.54–2.93)	1.80	2.48	3.10 (1.61–4.61)
Fiji	5.16	4.46	−2.11 (−6.78 to 2.80)	12.48	14.51	1.73 (1.45–2.02)	9.15	10.23	1.27 (0.95–1.58)	5.23	6.14	1.87 (1.51–2.24)
Marshall Islands	26.01	19.30	−4.80 (−6.81 to −2.74)	13.21	23.16	6.44 (6.32–6.55)	10.25	15.98	5.87 (4.50–7.25)	6.15	10.65	4.61 (2.37–6.90)
Tonga	38.27	15.11	−9.54 (−10.93 to −8.13)	39.44	56.16	3.97 (3.92–4.02)	29.22	41.28	3.85 (3.65–4.05)	16.02	23.04	4.19 (3.78–4.60)
Tuvalu	32.82	25.59	−2.50 (−5.10 to 0.17)	35.44	48.26	3.59 (3.46–3.72)	27.36	31.10	1.53 (1.30–1.76)	18.24	20.74	1.53 (1.30–1.76)
Kiribati	54.91	24.78	−7.89 (−18.68 to 4.34)	34.00	37.34	1.01 (0.85–1.16)	24.83	26.76	0.74 (0.46–1.02)	13.70	15.61	1.21 (0.80–1.62)
Micronesia, Federated States of	28.71	9.95	−7.26 (−12.97 to −1.17)	15.09	19.04	2.60 (2.48–2.72)	11.04	14.14	2.58 (1.98–3.19)	5.52	7.07	2.44 (1.17–3.71)
PNG	63.72	61.42	−0.16 (−1.57–1.28)	15.01	16.15	0.81 (0.77–0.85)	11.06	11.66	0.57 (0.54–0.60)	5.83	6.36	0.96 (0.92–1.01)
Solomon Islands	65.29	40.48	−6.90 (−10.24 to −3.44)	34.62	34.63	0.05 (−0.20 to 0.30)	25.81	25.88	0.08 (−0.19 to 0.35)	12.98	13.43	0.39 (0.10–0.68)
Samoa	14.73	11.30	−4.30 (−11.63 to 3.65)	12.49	14.93	1.96 (1.87–2.05)	9.42	11.01	1.81 (1.42–2.19)	4.96	5.96	2.05 (1.35–2.75)
Vanuatu	62.93	53.53	0.75 (−3.43 to 5.12)	34.61	36.21	0.51 (0.47–0.55)	25.55	26.54	0.42 (0.25–0.58)	13.15	14.03	0.83 (0.60–1.07)
Cook Islands	12.74	7.11	−6.83 (−10.71 to −2.78)	14.11	18.35	2.97 (2.89–3.05)	11.46	14.91	2.81 (2.51–3.11)	5.73	7.28	2.81 (2.51–3.11)
Niue	12.74	7.11	−6.83 (−10.71 to −2.78)	14.11	18.35	2.97 (2.89–3.05)	–	–	–	–	–	–
Tokelau	12.74	7.11	−6.83 (−10.71 to −2.78)	14.11	18.35	2.97 (2.89–3.05)	–	–	–	–	–	–
Wallis and Futuna Islands	12.74	7.11	−6.83 (−10.71 to −2.78)	14.11	18.35	2.97 (2.89–3.05)	8.22	8.87	0.81 (0.75–0.87)	8.22	8.87	0.81 (0.75–0.87)

EAPC = estimated annual percentage change in incidence (2015–2024), HBV = hepatitis B virus. HCC = hepatocellular carcinoma. DC = decompensated cirrhosis.

For consistency, all values were rounded to two decimal places. Values smaller than 0.01 are reported as <0.01. Countries without data reflect data unavailability or lack of modelled estimates, rather than the true absence of cases or services. Some identical or near-identical values across countries and areas reflect the use of regional comparator estimates within the CDA Foundation modelling framework, where country-specific empirical data were limited. These estimates should therefore be interpreted as modelled country-level values rather than independently observed measurements. HCC and DC EAPCs were not estimated for Niue and Tokelau because modelled rates were zero throughout 2015–2024 and could not be log-transformed. A declining trend was defined as an EAPC with a 95% CI entirely below zero, an increasing trend as an EAPC with a 95% CI entirely above zero, and no clear trend as an EAPC with a 95% CI that included zero.

HBV-related mortality followed a contrasting pattern. Mortality was classified as increasing in 31 of 34 countries and areas (91.2%), spanning all four analytical subregions. Japan (EAPC −3.09%, 95% CI −3.63 to −2.55) and New Zealand (−1.37%, 95% CI −1.51 to −1.24) had declining trends, while the Solomon Islands had no clear mortality trend (0.05%, 95% CI −0.20 to 0.30). The largest mortality increase occurred in the Marshall Islands (6.44%, 6.32 to 6.55), followed by Tonga and Cambodia (both 3.97%).

HCC and DC incidence generally increased alongside mortality, but varied between countries. Japan and New Zealand had declining trends in both HCC and DC incidence, and Nauru had declines in HCC and DC incidence despite increasing HBV-related mortality. The Marshall Islands had increases in mortality (EAPC 6.44%), HCC incidence (5.87%), and DC incidence (4.61%). EAPCs for HCC and DC could not be estimated for Niue and Tokelau as modelled rates were zero throughout the study period (Table 4). Residual diagnostics indicated evidence of positive serial correlation in 11 of 34 chronic incidence models and 30 of 34 mortality models; median lag-1 residual autocorrelation was 0.21 and 0.50, respectively.

### Country-level divergence between chronic HBV incidence and HBV-related mortality

Classification of country-specific trends by their EAPC 95% CIs showed frequent divergence between chronic HBV incidence and HBV-related mortality. Nineteen of 34 countries and areas (55.9%) were classified as having declining chronic HBV incidence alongside increasing mortality, while 12 (35.3%) had no clear incidence trend alongside increasing mortality (Fig. 2). Japan and New Zealand were the only two countries (5.9%) with concurrent declines in incidence and mortality, while Solomon Islands (2.9%) had declining incidence with no clear mortality trend. The declining-incidence/increasing-mortality pattern occurred across all four analytical subregions with no evidence that its frequency differed between them (Fisher's exact P = 0.185) (Fig. 2).

**Fig. 2:**
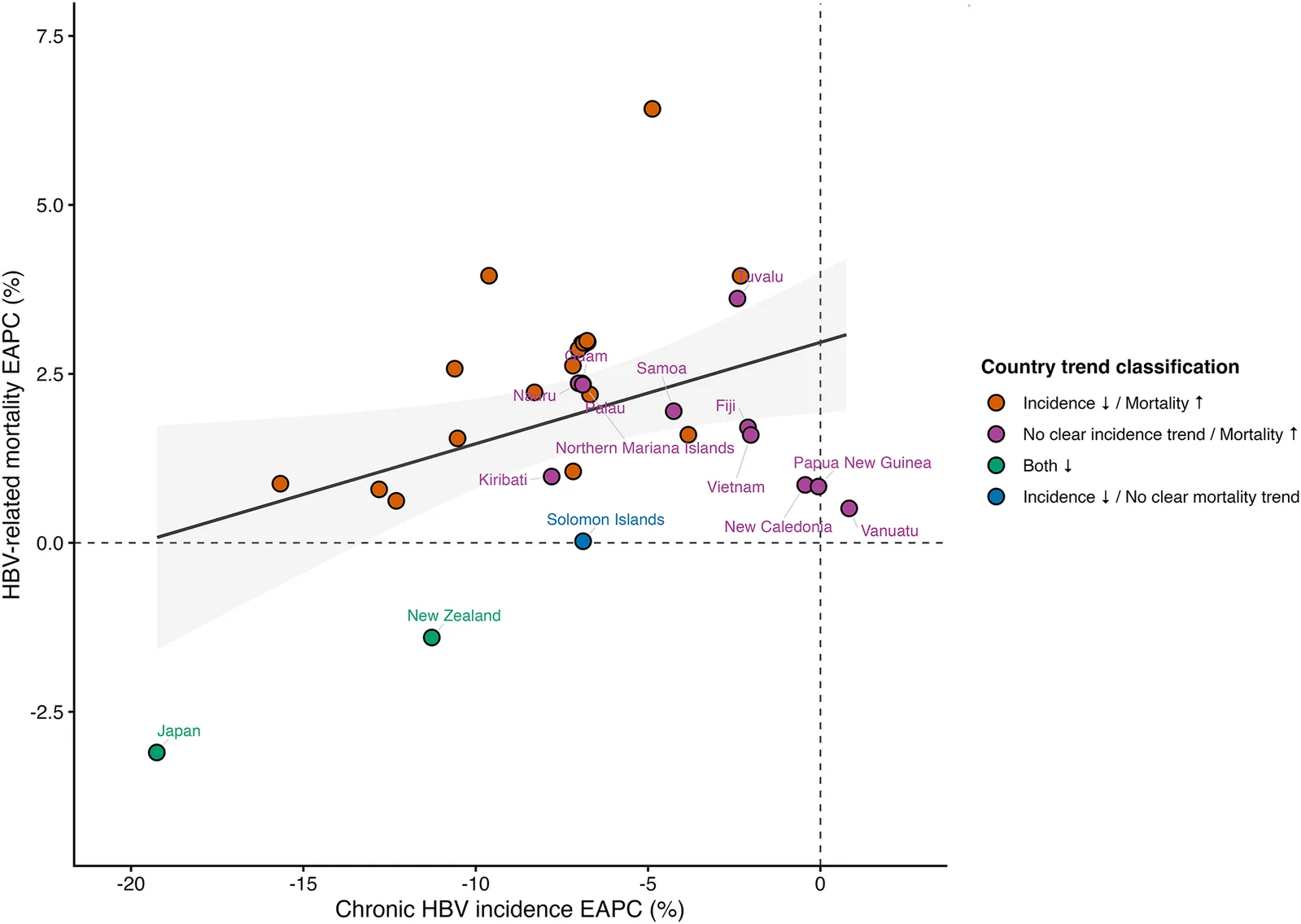
**Association between country-level trends in chronic HBV incidence and HBV-related mortality across 34 countries and areas in the WHO Western Pacific Region, 2015**–**2024.** Each point represents one country or area. Colours indicate combinations of chronic HBV incidence and HBV-related mortality trends classified using country-specific EAPCs and their corresponding 95% confidence intervals. A decreasing trend was defined as an EAPC with an upper 95% confidence limit below 0, an increasing trend as an EAPC with a lower 95% confidence limit above 0, and no clear trend when the 95% confidence interval included 0. The solid line represents the fitted linear regression and the shaded area represents the 95% confidence interval around the fitted regression line. Dashed reference lines indicate an EAPC of 0%. Several Pacific Island countries and areas share identical or near-identical model-derived estimates because regional comparator estimates were used where country-specific empirical data were limited; points were jittered slightly for visual separation only and jittering was not used in the regression analysis. EAPC = estimated annual percentage change. HBV = hepatitis B virus.

Country-level chronic HBV incidence and mortality EAPCs were modestly positively correlated (Pearson r = 0.39, 95% CI 0.06–0.64; P = 0.023) (Fig. 2). In simple linear regression, a one percentage-point higher chronic HBV incidence EAPC was associated with a 0.15 percentage-point higher mortality EAPC (β = 0.15; P = 0.023), with chronic HBV incidence EAPC explaining 15.2% of the between-country variation in mortality EAPC (R^2^ = 0.15).

The association was robust across three sensitivity analyses (Supplementary Table S5). Exclusion of the Marshall Islands, which had the highest mortality EAPC, produced similar estimates (n = 33; r = 0.40, 95% CI 0.06–0.65; β = 0.13; R^2^ = 0.16; P = 0.022) as did representing identical model-derived trajectories by single analytical observations (n = 27, r = 0.40, 95% CI 0.02–0.68; β = 0.15; R^2^ = 0.16; P = 0.039). Excluding PNG, New Caledonia, and Tuvalu, which showed substantial departures from log-linearity in chronic incidence, strengthened the association (n = 31; r = 0.49, 95% CI 0.16–0.72; β = 0.20; R^2^ = 0.24; P = 0.006) (Supplementary Table S5).

### Variation in HBV burden trends across analytical subregions

Despite the descriptive differences between subregions, formal comparisons provided limited evidence of systematic differences in country-level EAPCs across the four analytical subregions (Supplementary Table S2). Differences across subregions approached but did not reach statistical significance for chronic HBV incidence (Kruskal–Wallis χ^2^ = 7.47; P = 0.058) and mortality (χ^2^ = 7.56; P = 0.056). There was also no evidence of a difference in HCC incidence EAPCs across the four subregions (χ^2^ = 3.99; P = 0.263). DC incidence differed in the omnibus comparison (χ^2^ = 7.89; P = 0.048), but no pairwise comparison remained statistically significant after Holm adjustment (Supplementary Table S2).

### Vaccination coverage remained high overall but varied substantially between countries

At the regional level, HepB3 coverage remained above 90% throughout most of the study period, whereas HepB-BD coverage remained approximately 80% and below the 2030 target of ≥90% (Fig. 1). Country-level vaccination coverage varied across analytical subregions (Table 3, Fig. 3). In 2024, HepB3 coverage was at least 95% in all WEA countries, while HepB-BD coverage exceeded 90% in China, the Republic of Korea, and Mongolia; Japan and New Zealand used targeted rather than universal birth-dose strategies. Coverage was more variable in WSEA, where 2024 HepB3 ranged from 64.90% in Viet Nam to 99.93% in Brunei Darussalam and HepB-BD from 57.37% in the Philippines to 99.00% in Malaysia. Variation was also pronounced among PICTs with available vaccination data. In 2024, HepB-BD coverage ranged from 26.20% in PNG to 99.65% in Tuvalu, and HepB3 coverage ranged from 39.84% in PNG to 99.17% in Tonga. PNG had persistently low coverage, with HepB-BD declining from 32.00% in 2015 to 26.20% in 2024 and HepB3 from 62.00% to 39.84%. Fiji maintained HepB3 coverage above 90%, but HepB-BD coverage declined from 99.00% in 2020 to 39.12% in 2024. Declines in HepB3 coverage were also seen in several PICTs, including the Cook Islands (from 99.00% in 2015 to 69.00% in 2024) and Samoa (from 94.00% to 83.00% in 2024). Vaccination data were unavailable for six countries and areas (Table 3, Fig. 3; Supplementary Table S1).

**Fig. 3:**
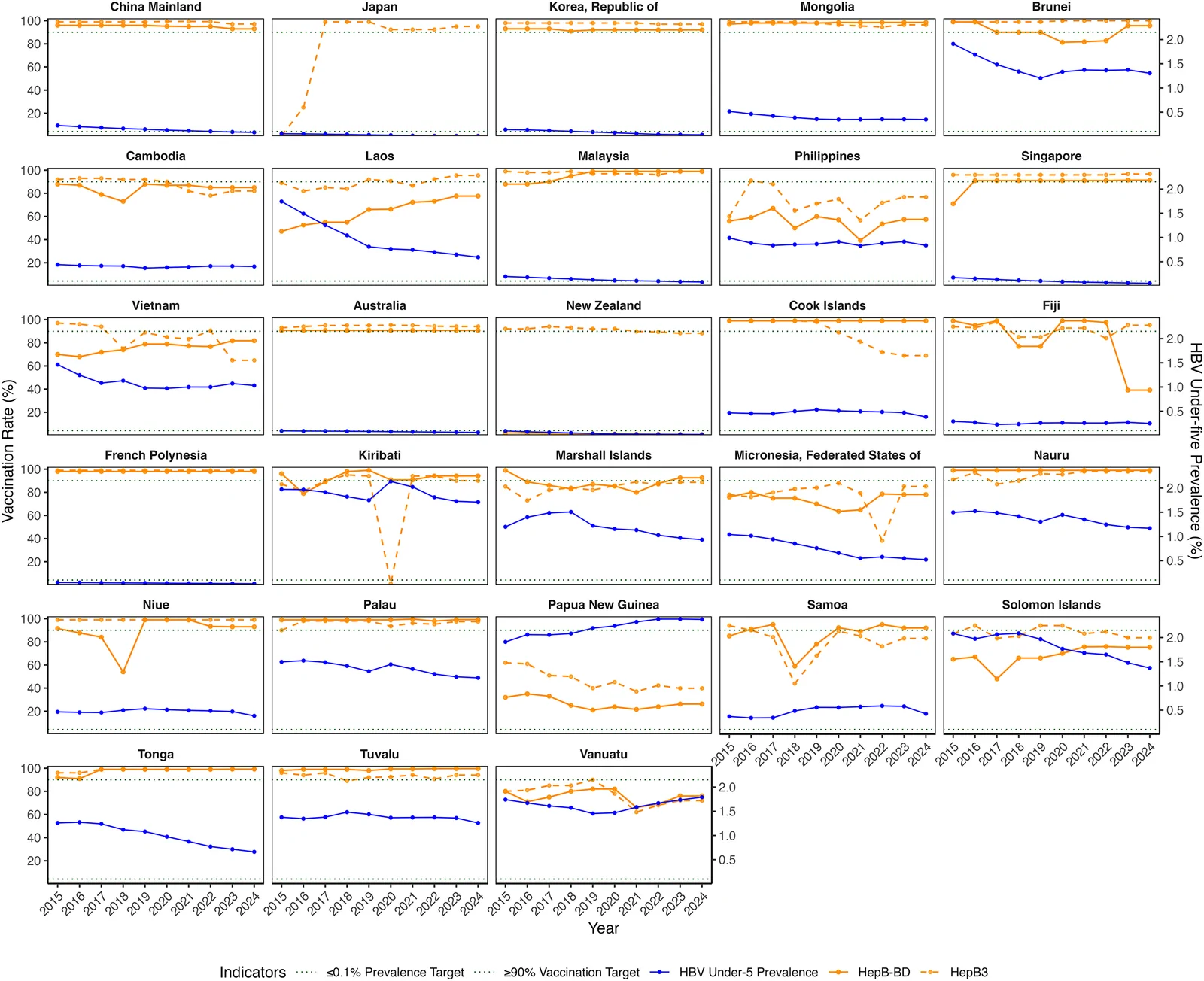
**Country-level trends in hepatitis B vaccination coverage and under-five HBV prevalence in the WPR, 2015**–**2024.** Country-level trends in hepatitis B birth-dose vaccination coverage (HepB-BD), hepatitis B three-dose vaccination coverage (HepB3), and under-five HBV prevalence from 2015 to 2024 across countries and areas in the Western Pacific Region. In each panel, HepB-BD and HepB3 coverage are shown on the left y-axis and under-five HBV prevalence on the right y-axis. Horizontal reference lines indicate the WHO 2030 targets: ≥90% coverage for HepB-BD and HepB3, and ≤0.1% under-five HBV prevalence. HBV = hepatitis B virus. HepB-BD = hepatitis B birth dose. HepB3 = hepatitis B three-dose vaccination coverage. WPR = Western Pacific Region.

### Exploratory associations between vaccination coverage and changes in HBV burden

Among 28 countries and areas with HepB-BD and HepB3 coverage data in both 2015 and 2024, neither baseline coverage nor changes in coverage showed robust associations with changes in under-five HBV prevalence or chronic HBV incidence EAPCs after adjustment for multiple comparisons (Supplementary Table S3). The strongest observed association was between changes in HepB3 coverage and chronic HBV incidence EAPC (Spearman ρ = −0.44; unadjusted P = 0.02); however, this association did not remain statistically significant after Holm adjustment (adjusted P = 0.17). Excluding Japan, which introduced HepB3 vaccination into its national immunisation programme in 2016, attenuated the association while retaining its inverse direction (ρ = −0.37; P = 0.057; Supplementary Table S3).

### Progress towards WHO interim milestones and elimination targets was uneven

Progress towards WHO interim milestones and elimination targets remained uneven (Table 1; Supplementary Table S1; Fig. 4, Fig. 5, Fig. 6, Fig. 7). By 2024, all WEA and Australasia countries had reached at least the 2025 interim milestone for under-five prevalence, with China, the Republic of Korea, Japan, Australia, New Zealand and French Polynesia reaching the 2030 target of ≤0.1%. Progress was less uniform in WSEA and the PICTs; Brunei Darussalam, Viet Nam and several PICTs remained above 1.0%, with the highest prevalence seen in PNG (2.38%) and New Caledonia (2.17%) (Supplementary Table S1).

**Fig. 4:**
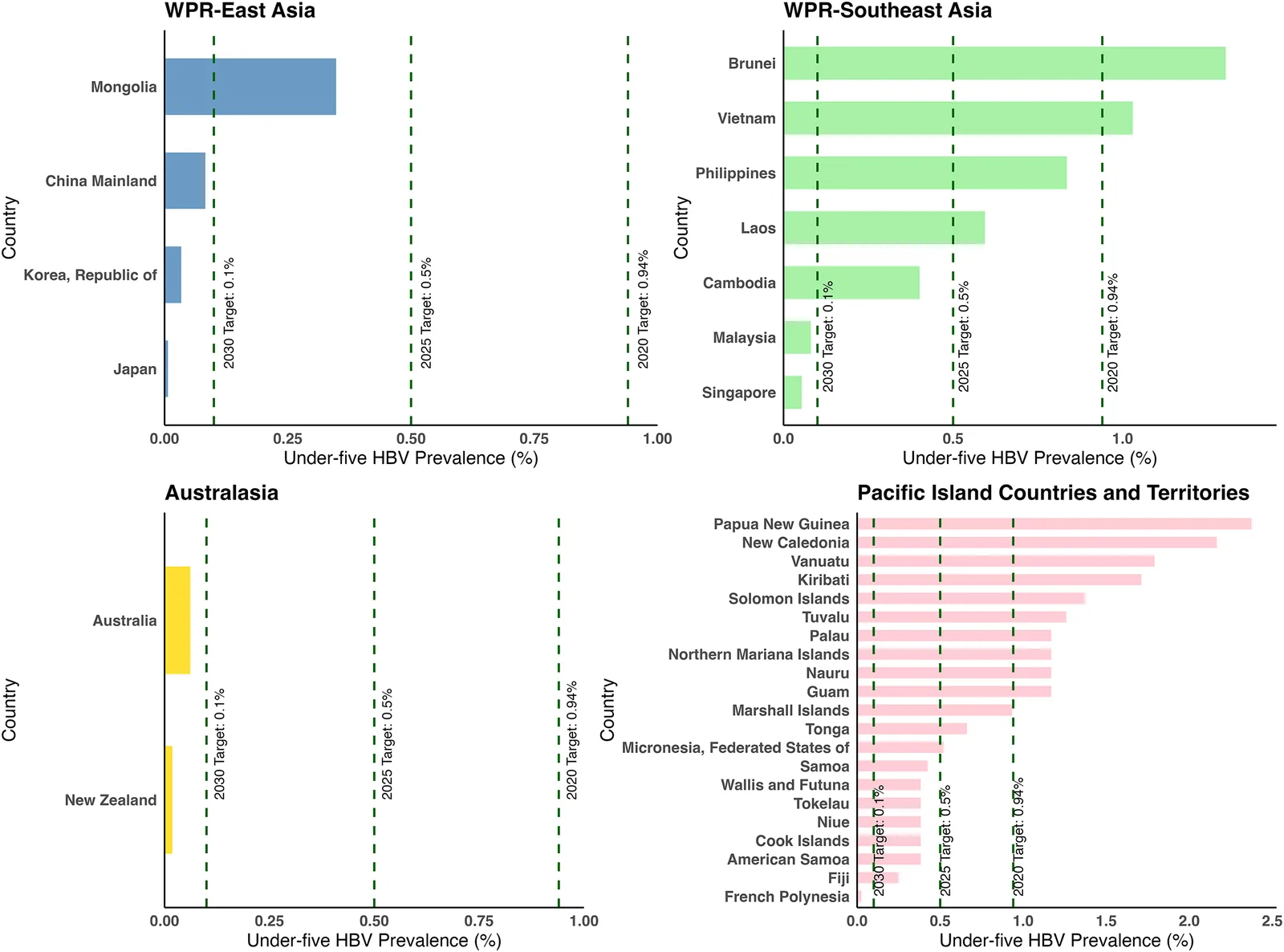
**Progress towards WHO under-five HBV prevalence milestones and targets across WPR countries, 2024.** Under-five HBV prevalence (%) in 2024 across countries and areas in the Western Pacific Region, grouped by analytical subregion. Vertical reference lines indicate the WHO 2020 baseline (0.94%), 2025 interim milestone (0.5%), and 2030 elimination target (0.1%). HBV = hepatitis B virus. WPR = Western Pacific Region.

**Fig. 5:**
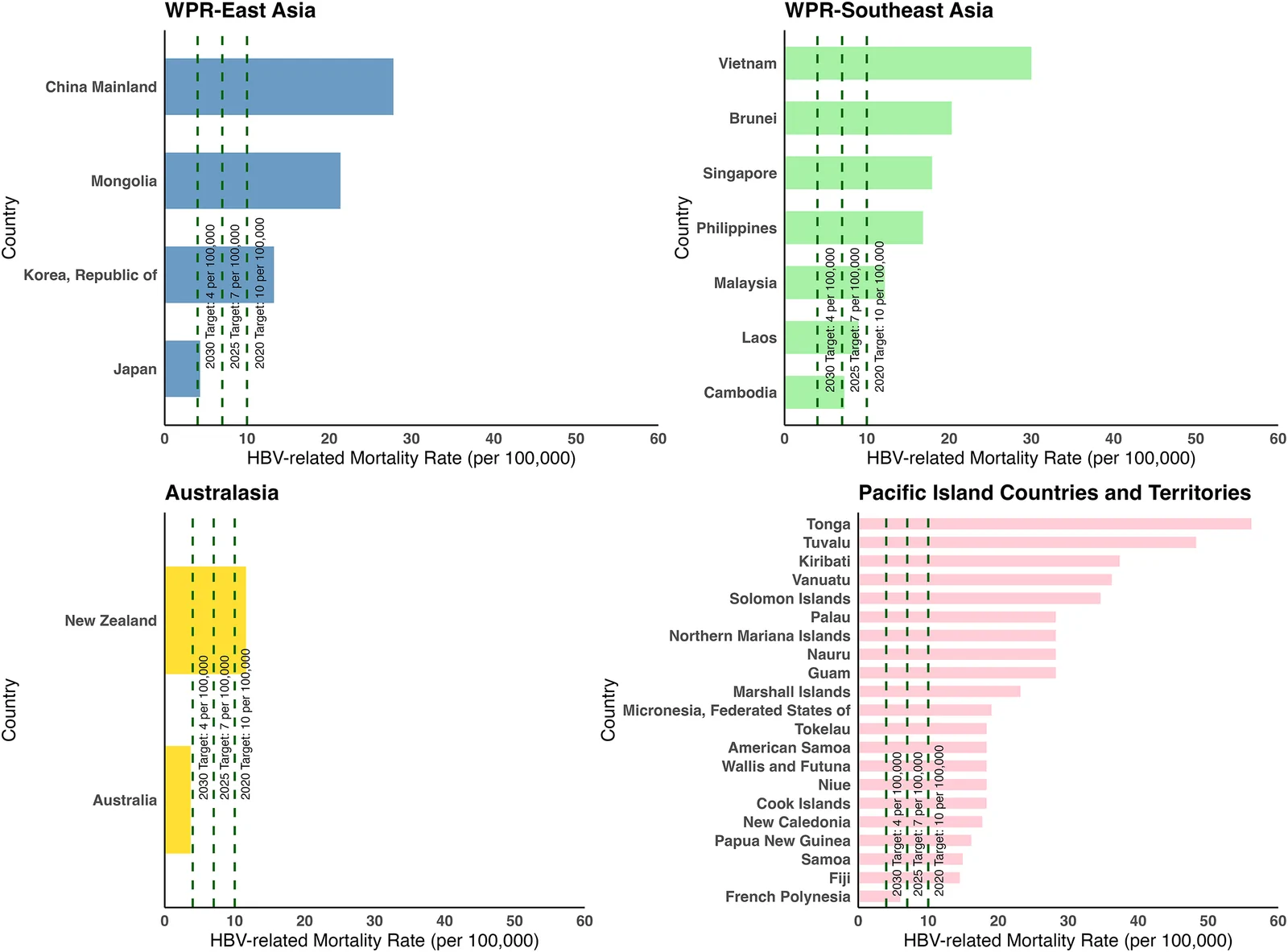
**Progress towards WHO HBV-related mortality milestones and targets across WPR countries, 2024.** HBV-related mortality rate per 100,000 population in 2024 across countries and areas in the Western Pacific Region, grouped by analytical subregion. Vertical reference lines indicate the WHO 2020 baseline (10 per 100,000 population), 2025 interim milestone (7 per 100,000 population), and 2030 elimination target (4 per 100,000 population). HBV = hepatitis B virus. WPR = Western Pacific Region.

**Fig. 6:**
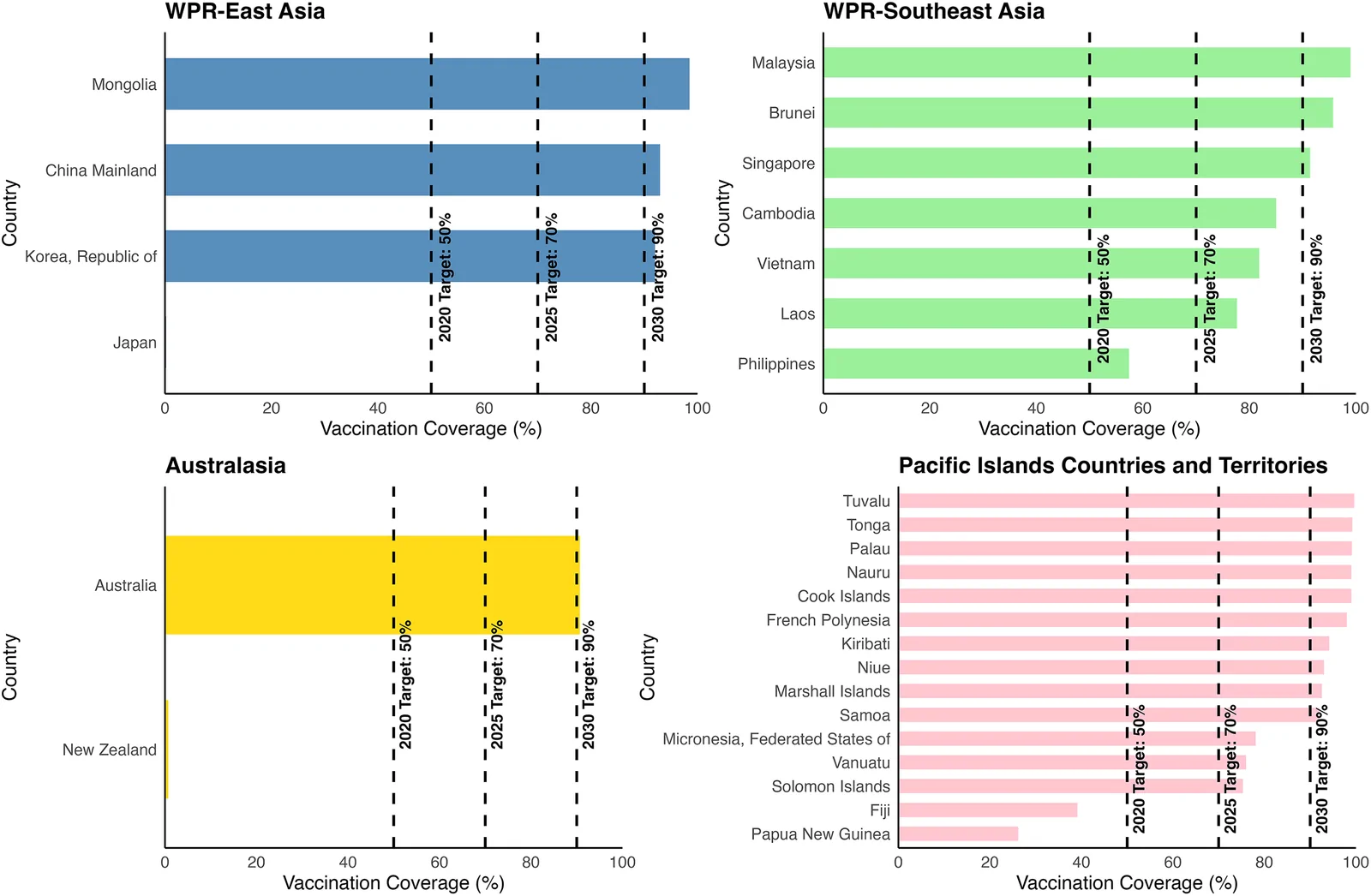
**Progress towards WHO HepB-BD coverage milestones and targets across WPR countries, 2024.** Hepatitis B birth-dose vaccination coverage (HepB-BD, %) in 2024 across countries and areas in the Western Pacific Region, grouped by analytical subregion. Vertical reference lines indicate the WHO 2020 baseline (50%), 2025 interim milestone (70%), and 2030 elimination target (90%). HepB-BD = hepatitis B birth dose. WPR = Western Pacific Region.

**Fig. 7:**
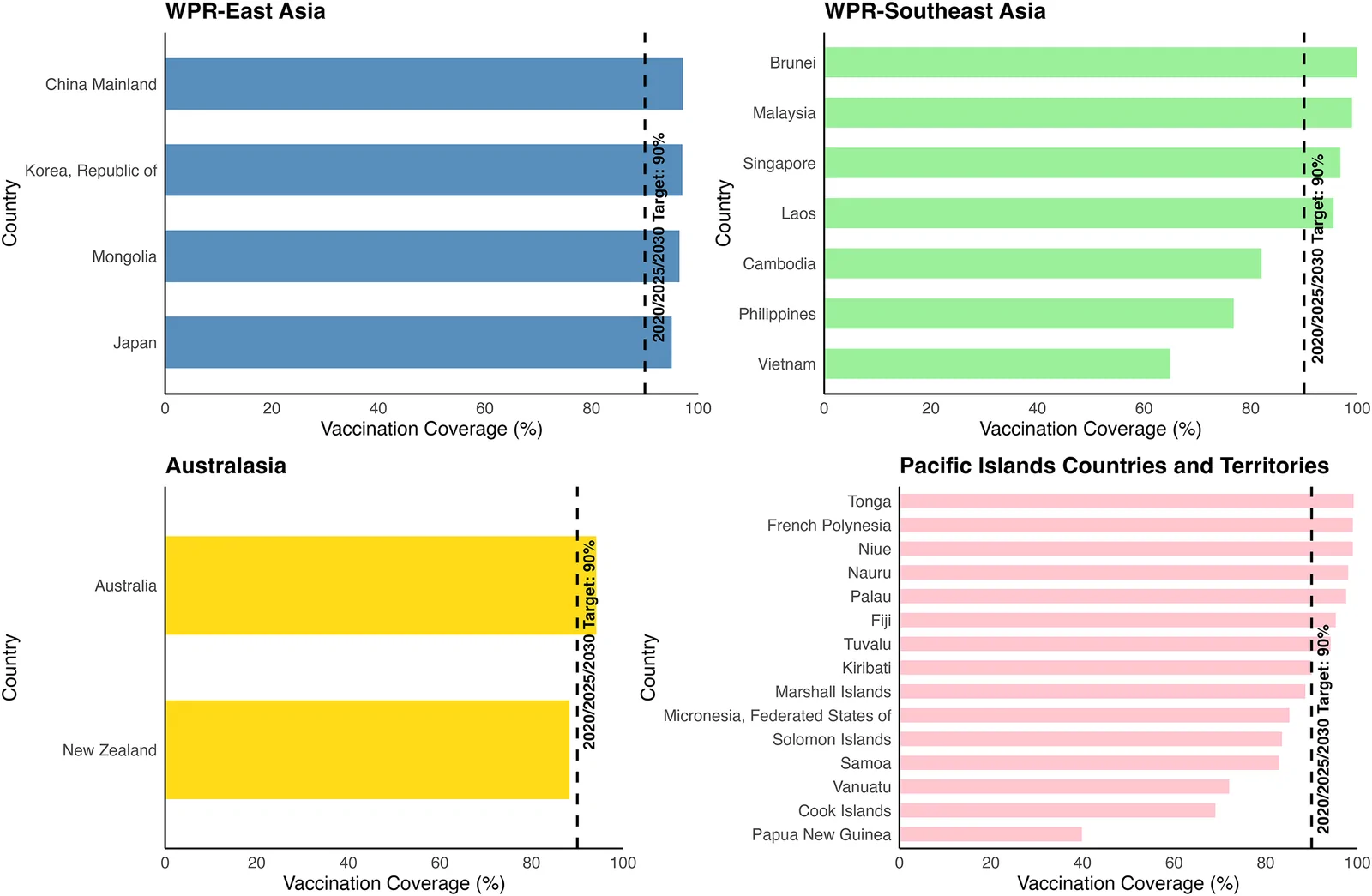
**Progress towards WHO HepB3 coverage milestones and targets across WPR countries, 2024.** Hepatitis B three-dose vaccination coverage (HepB3, %) in 2024 across countries and areas in the Western Pacific Region, grouped by analytical subregion. Vertical reference lines indicate the WHO 2020 baseline (90%), 2025 interim milestone (90%), and 2030 elimination target (90%). HepB3 = hepatitis B three-dose vaccination coverage. WPR = Western Pacific Region.

Progress toward the mortality target was substantially more limited. In 2024, Australia (3.70 per 100,000), Japan (4.29 per 100,000) and French Polynesia (5.99 per 100,000) were the only countries below the 2025 interim threshold of 7 per 100,000, and Australia alone had reached the 2030 target of ≤4 per 100,000. Vaccination milestones were more widely achieved but remained heterogeneous, with substantial gaps in several WSEA and Pacific settings, particularly PNG (HepB-BD 26.20%; HepB3 39.84% in 2024) (Supplementary Table S1; Fig. 4, Fig. 5, Fig. 6, Fig. 7).

### Sensitivity analysis using income classification

To assess whether the findings from the analytical subregional grouping were sensitive to an alternative country classification, EAPCs were compared using World Bank FY2025 income groups. There was no evidence of differences across lower-middle-income, upper-middle-income, and high-income countries and areas for chronic HBV incidence (P = 0.227), HBV-related mortality (P = 0.597), HCC incidence (P = 0.625), or DC incidence (P = 0.150) (Supplementary Table S4). Four countries and areas without an FY2025 World Bank income classification were excluded from this analysis.

## Discussion

This ecological analysis extends previous regional descriptions of HBV burden by quantifying country-specific temporal trajectories and examining whether changes in chronic HBV incidence and mortality occurred in parallel across the WPR. Although prevalence and chronic incidence declined during 2015–2024, they were frequently accompanied by increasing mortality: 55.9% of countries and areas had declining incidence alongside increasing mortality, whereas only Japan and New Zealand were classified as declining in both. Incidence and mortality EAPCs were modestly correlated, with incidence trends accounting for 15.2% of between-country variation in mortality trends. These findings demonstrate divergent trajectories across individual WPR countries and areas. Contextual comparison with the WHO 2026 assessment and independent GBD 2021 estimates showed broadly consistent patterns of declining incidence alongside persistent or increasing absolute mortality burden (Supplementary Table S6).^1^^,^^16^

The observed divergence does not establish why mortality has continued to increase. Chronic HBV can progress over decades to cirrhosis, hepatic decompensation, and HCC, such that contemporary mortality can reflect infections acquired long before recent reductions in transmission.^4^^,^^17^ This temporal disconnect has also been described across the Asia–Pacific, where population ageing may contribute.^18^ Independent GBD 2021 estimates showed increasing absolute chronic HBV deaths despite declining age-standardised mortality, with mortality increasingly concentrated at older ages. Demographic expansion and ageing have also contributed to rising HBV-attributable liver cancer deaths.^16^^,^^19^ Diagnosis and treatment uptake, liver cancer surveillance, changes in ascertainment, and competing causes of death could also influence mortality.^1^ These mechanisms were not directly evaluated in this study, and their relative contributions cannot be determined. Nevertheless, the modest association between incidence and crude modelled mortality EAPCs suggests that contemporary incidence trends alone provide limited information about country-level mortality trajectories. Prevention of new infections and reduction of deaths should therefore be considered related but temporally distinct dimensions of elimination progress.

Prevention remains central to HBV elimination, but national vaccination coverage did not fully explain contemporary differences in childhood infection or chronic incidence across the WPR. Although under-five prevalence declined substantially, it plateaued at approximately 0.28–0.29% from 2019 and remained above the WHO 2030 target. Neither baseline HepB-BD or HepB3 coverage nor changes in coverage showed robust associations with changes in under-five prevalence or chronic incidence after adjustment for multiple comparisons. This does not challenge established vaccine effectiveness, but highlights the limitations of contemporary national coverage as a proxy for cumulative prevention performance. HepB-BD coverage also does not capture administration within 24 h of birth or other components of prevention of mother-to-child transmission.^18^ As countries approach elimination, residual childhood infection may increasingly depend on timely birth-dose delivery, maternal screening, and antiviral prophylaxis for pregnant women at high risk of transmission. WHO's 2024 guidelines emphasise these interventions and simplified diagnostic pathways.^20^ HBIG coverage and maternal antiviral uptake were not included in this analysis, precluding assessment of their contribution to the observed heterogeneity.

WHO's 2026 assessment identified a disproportionate share of global HBV mortality and substantial remaining diagnosis and treatment gaps in the WPR,^1^ consistent with documented care-cascade gaps across the Asia-Pacific.^18^ Achievement of prevention and transmission targets therefore cannot be assumed to indicate comparable progress towards the mortality target. As vaccination programmes mature, reducing near-term complications and deaths increasingly requires clinical management of existing chronic infection. Antiviral treatment can reduce progression to cirrhosis and HCC and improve survival, while current WHO guidance has expanded treatment eligibility and simplified diagnosis and long-term care.^20^ Elimination monitoring should therefore assess prevention, incidence, care-cascade performance, and mortality as complementary outcomes.

Barriers to HBV elimination differ substantially across the WPR. Where childhood infection and chronic incidence have fallen, but mortality has not, priorities increasingly include managing established chronic infection while sustaining prevention. Countries with persistent childhood infection face the dual challenge of preventing transmission and managing existing disease. This distinction is particularly relevant in the Pacific, where neither analytical subregion nor income classification adequately explained the observed heterogeneity. Instead, persistent childhood burden in particular settings points towards country-specific programme gaps. Studies in PNG have documented barriers to timely HepB-BD delivery, including births outside health facilities, delayed presentation, vaccine availability, cold-chain constraints, and implementation challenges.^21^^,^^22^ Continuing vertical transmission has also been reported in Kiribati despite longstanding vaccination programmes.^23^ Thus, high regional vaccination coverage cannot establish interruption of mother-to-child and early-childhood transmission within individual countries. National programmes need to identify which components of prevention and care remain limiting within their own settings.

Countries with successful transmission control but large populations with longstanding chronic HBV face a different challenge. China exemplifies this transition. Despite major reductions in childhood infection, an estimated 75 million people remain chronically infected, with substantial diagnosis and treatment gaps and an increasing concentration of infection among older cohorts.^24^ Similar patterns occur across the Asia–Pacific, where declining new infections coexist with substantial populations at risk of cirrhosis and HCC.^18^ This context is consistent with our finding of declining chronic incidence alongside increasing modelled mortality in China, although it does not establish causation. Countries advanced in transmission control must therefore sustain prevention while increasingly addressing diagnosis, treatment, and long-term outcomes among people with chronic HBV.

This study has several strengths and limitations. Strengths include a common modelling framework across 34 WPR countries and areas, formal quantification of country-specific temporal trends, and sensitivity analyses addressing influential observations, shared model-derived trajectories, departures from log-linearity, and alternative country classification. However, the modelled estimates should not be interpreted as equivalent to directly observed surveillance data. The availability, quality, and recency of empirical inputs vary between countries, particularly in small-island settings, where model assumptions and small absolute changes may have greater influence on estimated trends. Uncertainty intervals were unavailable for incidence, mortality, HCC, and DC; therefore, EAPC confidence intervals represent uncertainty around the fitted trends but not the underlying modelled estimates. Residual autocorrelation was evident particularly for mortality trajectories, and temporal dependence or smoothing of annual model-derived estimates could result in overly precise conventional regression confidence intervals. Country-level EAPC classifications should therefore be interpreted cautiously. The ecological design precludes causal inference about relationships between vaccination, incidence, mortality, and health-system factors. Age-standardised mortality rates and individual-level care data were unavailable, while country-level diagnosis and treatment indicators were outside the scope of this analysis. HBIG coverage, maternal antiviral uptake, and other determinants of residual transmission and mortality were not assessed. The analytical subregions should not be interpreted as epidemiologically homogeneous groups, however, sensitivity analysis using World Bank income classification similarly provided little evidence that broad country groupings explained the observed heterogeneity.

In conclusion, declining HBV incidence has not been accompanied by declining mortality in many WPR countries and areas, with substantial heterogeneity in both transmission and disease burden. Regional progress and prevention indicators alone are therefore insufficient to characterise elimination progress. Monitoring should assess transmission, care-cascade performance, and mortality alongside vaccination, with priorities determined by country-specific gaps. Better age-specific mortality, diagnosis, treatment, and prevention-of-mother-to-child-transmission data are needed to explain divergent trajectories and validate modelled trends. Simplified diagnostic approaches, including point-of-care HBV DNA testing, could further improve access to assessment and treatment where laboratory capacity is limited.^25^^,^^26^

## Contributors

**Hsu Thinzar Maung:** conceptualisation, study design, data curation, literature search, formal analysis, data verification, data interpretation, visualisation, writing of the original draft, review and editing of the manuscript, project administration; **Mohamed Amine Ghrabi:** data curation, data verification, data interpretation, conceptualisation, review and editing of the manuscript; **Fukushi Morishita:** project administration, data interpretation, review and editing of the manuscript; **Rajendra Prasad Hubraj Yadav:** data interpretation, review and editing of the manuscript; **Huong Thi Giang Tran:** data interpretation, review and editing of the manuscript; **Kiyohiko Izumi:** supervision, project administration, study design, conceptualisation, data curation, data verification, data interpretation, review and editing of the manuscript.

HTM and KI accessed and verified the underlying data. All authors reviewed the manuscript, approved the final version, and accepted responsibility for the decision to submit for publication.

## Data sharing statement

Selected headline hepatitis B indicators are publicly available through the Polaris Observatory of the Center for Disease Analysis Foundation. The analytical dataset used in this study was a separate country-level extract covering 2015–2024 and included additional modelled indicators not available in the public database. Access to these non-public data is subject to the data-sharing policies and permissions of the Center for Disease Analysis Foundation and is not controlled by the authors. Requests for access to non-public data should therefore be directed to the Center for Disease Analysis Foundation. The R code used for the statistical analyses, sensitivity analyses, and figure generation will be made available by the corresponding author upon reasonable request.

## Declaration of generative AI and AI-assisted technologies in the manuscript preparation process

During the preparation of this work, the author (s) used Microsoft Copilot and Grammarly for language editing, paraphrasing, and manuscript formatting. The author(s) reviewed and edited the output as needed and take full responsibility for the content of the published article.

## Declaration of interests

We declare no competing interests.
