# Supplementary material for: Trends and disparities in hepatitis B burden in the Western Pacific Region, 2015–2024: a subregional analysis using modelled data
Source: Lancet Reg Health West Pac. 2026 Sep 24;75:101991. doi: 10.1016/j.lanwpc.2026.101991 (PMC13634976; doi:10.1016/j.lanwpc.2026.101991)
Supplement: Supplementary file 1 [file mmc1.docx]

# Supplementary Appendix

Trends and Disparities in Hepatitis B Burden in the Western Pacific Region, 2015-2024: A Subregional Analysis Using Modelled Data

**Authors**

Hsu Thinzar Maung ^1,2*^, Mohamed Amine Ghrabi^1^, Fukushi Morishita^1^, Rajendra Prasad Hubraj Yadav^1^, Huong Thi Giang Tran^1^ and Kiyohiko Izumi^1^

Table of Contents

[Supplementary Appendix	1](#_Toc237708783)

[1.	Statement of GATHER compliance	3](#_Toc237708784)

[2.	Supplementary Tables	4](#_Toc237708785)

[Supplementary Table 1. Progress towards WHO 2025 interim hepatitis B elimination milestones in WPR countries: 2015, 2020, and 2024	4](#_Toc237708786)

[Supplementary Table 2. Comparison of country-level EAPCs in HBV burden indicators across analytical subregions, 2015–2024	6](#_Toc237708787)

[Supplementary Table 3. Exploratory associations between hepatitis B vaccination coverage and changes in modelled HBV burden across countries and areas with available vaccination data, 2015–2024	7](#_Toc237708788)

[Supplementary Table 4. Sensitivity analysis of country-level HBV burden trends according to World Bank FY2025 income classification, 2015–2024	8](#_Toc237708789)

[Supplementary Table 5. Sensitivity analyses of the association between estimated annual percentage changes in chronic HBV incidence and HBV-related mortality, 2015–2024	9](#_Toc237708790)

[Supplementary Table 6. Comparison of hepatitis B burden estimates from the study analytical dataset and the WHO Global hepatitis report 2026, Western Pacific Region	10](#_Toc237708791)

# 1. Statement of GATHER compliance

This study reports modelled country-level HBV estimates derived from CDA Foundation data for 34 countries and areas in the Western Pacific Region from 2015 to 2024. The main manuscript describes the data source, modelling framework, geographic scope, inclusion and exclusion criteria, outcomes, analytical approach, uncertainty reporting, limitations, and data-sharing arrangements in accordance with GATHER principles.

# 2. Supplementary Tables

## Supplementary Table 1. Progress towards WHO 2025 interim hepatitis B elimination milestones in WPR countries: 2015, 2020, and 2024

| **Country** | **Impact indicator** | | | | | | **Service coverage** | | | | | |
| --- | --- | --- | --- | --- | --- | --- | --- | --- | --- | --- | --- | --- |
|  | **Under-five HBV prevalence (%)** | | | **HBV-related mortality rate**  **(per 100 000)** | | | **HepB-BD coverage (%)** | | | **HepB3 coverage**  **(%)** | | |
|  | **2015** | **2020** | **2024** | **2015** | **2020** | **2024** | **2015** | **2020** | **2024** | **2015** | **2020** | **2024** |
| **WEA** | | | | | | | | | | | | |
| **China** | 0·23 | 0·13 | 0·08 | 25·89 | 26·92 | 27·79 | 96·00 | 95·23 | 92·95 | 99·00 | 99·38 | 97·10 |
| **Republic of Korea** | 0·14 | 0·07 | 0·03 | 12·28 | 12·73 | 13·28 | 93·00 | 92·00 | 92·00 | 98·00 | 98·00 | 97·00 |
| **Japan** | 0·05 | 0·02 | 0·01 | 5·73 | 4·63 | 4·29 | 0·43 | 0·31 | 0·23 | 0·43 | 92·33 | 95·00 |
| **Mongolia** | 0·52 | 0·35 | 0·35 | 17·56 | 19·39 | 21·36 | 97·00 | 98·50 | 98·51 | 99·00 | 96·40 | 96·46 |
| **WSEA** | | | | | | | | | | | | |
| **Singapore** | 0·17 | 0·09 | 0·05 | 14·95 | 17·03 | 17·93 | 71·00 | 91·00 | 91·40 | 96·00 | 96·00 | 96·80 |
| **Brunei Darussalam** | 1·91 | 1·33 | 1·30 | 17·64 | 19·14 | 20·31 | 99·00 | 81·31 | 95·70 | 99·00 | 99·97 | 99·93 |
| **Malaysia** | 0·20 | 0·11 | 0·08 | 11·52 | 11·94 | 12·17 | 88·00 | 99·00 | 99·00 | 99·00 | 97·00 | 99·00 |
| **Cambodia** | 0·44 | 0·38 | 0·40 | 5·12 | 6·29 | 7·26 | 88·00 | 87·00 | 85·00 | 92·00 | 90·00 | 82·00 |
| **Lao PDR** | 1·74 | 0·76 | 0·59 | 7·84 | 8·37 | 8·99 | 47·11 | 66·28 | 77·65 | 89·00 | 90·68 | 95·52 |
| **Viet Nam** | 1·46 | 0·97 | 1·03 | 26·06 | 27·86 | 29·99 | 70·00 | 79·00 | 81·82 | 97·00 | 85·00 | 64·90 |
| **Philippines** | 0·99 | 0·91 | 0·84 | 13·10 | 15·06 | 16·82 | 56·00 | 57·00 | 57·37 | 60·00 | 75·00 | 76·81 |
| **Australasia** | | | | | | | | | | | | |
| **Australia** | 0·09 | 0·08 | 0·06 | 3·35 | 3·52 | 3·70 | 90·72 | 90·72 | 90·72 | 93·00 | 95·33 | 94·11 |
| **New Zealand** | 0·09 | 0·03 | 0·02 | 13·12 | 12·07 | 11·60 | 2·10 | 1·24 | 0·71 | 92·08 | 92·08 | 88·29 |
| **PICTs** | | | | | | | | | | | | |
| **American Samoa** | 0·47 | 0·51 | 0·38 | 14·11 | 16·45 | 18·35 | .. | .. | .. | .. | .. | .. |
| **Guam** | 1·50 | 1·45 | 1·17 | 22·81 | 26·06 | 28·21 | .. | .. | .. | .. | .. | .. |
| **Northern Mariana Islands** | 1·50 | 1·45 | 1·17 | 22·81 | 26·06 | 28·21 | .. | .. | .. | .. | .. | .. |
| **New Caledonia** | 1·81 | 2·06 | 2·17 | 16·47 | 17·21 | 17·75 | .. | .. | .. | .. | .. | .. |
| **Nauru** | 1·50 | 1·45 | 1·17 | 22·81 | 26·06 | 28·21 | 99·00 | 99·00 | 99·00 | 91·00 | 95·43 | 97·98 |
| **Palau** | 1·50 | 1·45 | 1·17 | 22·81 | 26·06 | 28·21 | 99·00 | 99·00 | 99·12 | 90·00 | 93·48 | 97·51 |
| **French Polynesia** | 0·05 | 0·04 | 0·03 | 4·77 | 5·54 | 5·99 | 98·00 | 98·00 | 98·00 | 99·00 | 99·00 | 99·00 |
| **Fiji** | 0·29 | 0·26 | 0·25 | 12·48 | 13·96 | 14·51 | 99·00 | 99·00 | 39·12 | 94·00 | 92·77 | 95·24 |
| **Marshall Islands** | 1·20 | 1·16 | 0·93 | 13·21 | 17·89 | 23·16 | 99·00 | 85·36 | 92·54 | 85·00 | 85·67 | 88·62 |
| **Tonga** | 1·26 | 0·98 | 0·66 | 39·44 | 48·00 | 56·16 | 92·00 | 99·00 | 99·20 | 96·00 | 99·00 | 99·17 |
| **Tuvalu** | 1·38 | 1·37 | 1·26 | 35·44 | 41·82 | 48·26 | 98·00 | 99·39 | 99·65 | 96·00 | 92·56 | 94·14 |
| **Kiribati** | 1·97 | 2·14 | 1·71 | 34·00 | 36·46 | 37·34 | 96·00 | 90·72 | 94·16 | 87·00 | 0·96 | 90·00 |
| **Micronesia, Federated States of** | 1·04 | 0·66 | 0·52 | 15·09 | 17·38 | 19·04 | 76·00 | 63·54 | 78·10 | 78·00 | 87·86 | 85·13 |
| **PNG** | 1·91 | 2·24 | 2·38 | 15·01 | 15·68 | 16·15 | 32·00 | 23·76 | 26·20 | 62·00 | 45·20 | 39·84 |
| **Solomon Islands** | 2·08 | 1·77 | 1·37 | 34·62 | 33·98 | 34·63 | 65·00 | 70·00 | 75·29 | 87·00 | 94·00 | 83·51 |
| **Samoa** | 0·37 | 0·56 | 0·43 | 12·49 | 13·83 | 14·93 | 85·00 | 92·23 | 92·00 | 94·00 | 89·24 | 83·00 |
| **Vanuatu** | 1·74 | 1·47 | 1·79 | 34·61 | 35·62 | 36·21 | 80·00 | 82·00 | 76·00 | 80·00 | 78·00 | 72·00 |
| **Cook Islands** | 0·47 | 0·51 | 0·38 | 14·11 | 16·45 | 18·35 | 99·00 | 99·00 | 98·99 | 99·00 | 89·00 | 69·00 |
| **Niue** | 0·47 | 0·51 | 0·38 | 14·11 | 16·45 | 18·35 | 91·50 | 99·00 | 93·00 | 99·00 | 99·00 | 99·00 |
| **Tokelau** | 0·47 | 0·51 | 0·38 | 14·11 | 16·45 | 18·35 | .. | .. | .. | .. | .. | .. |
| **Wallis and Futuna Islands** | 0·47 | 0·51 | 0·38 | 14·11 | 16·45 | 18·35 | .. | .. | .. | .. | .. | .. |

Colour categories indicate performance relative to WHO 2025 interim milestones. For consistency, all values were rounded to two decimal places. Japan and New Zealand implement targeted birth dose strategies rather than universal HepB-BD vaccination. Japan introduced HepB3 vaccination into the national immunisation program in October 2016. Countries without data reflect data unavailability or lack of modelled estimates, rather than the true absence of cases or services. Some identical or near-identical values across countries and areas reflect the use of regional comparator estimates within the CDA Foundation modelling framework, where country-specific empirical data were limited. These estimates should therefore be interpreted as modelled country-level values rather than independently observed measurements. For under-five HBV prevalence, green indicates ≤0·5%, yellow >0·5% to ≤1·0%, and red >1·0%. For HBV-related mortality, green indicates ≤7 per 100 000, yellow >7 to ≤10, and red >10. For HepB-BD coverage, green indicates ≥70%, yellow ≥50% to <70%, and red <50%. For HepB3 coverage, green indicates ≥90%, yellow ≥70% to <90%, and red <70%.

## Supplementary Table 2. Comparison of country-level EAPCs in HBV burden indicators across analytical subregions, 2015–2024

| **Indicator** | **Australasia** | **WEA** | **PICTs** | **WSEA** | **χ²** | **P value** |
| --- | --- | --- | --- | --- | --- | --- |
| Chronic incidence | −9·30 (2·07) | −14·30 (5·23) | −6·83 (2·63) | −7·05 (6·33) | 7·47 | 0·058 |
| HBV mortality | −0·16 (1·22) | 0·85 (1·40) | 2·37 (1·23) | 1·58 (0·96) | 7·56 | 0·056 |
| HCC incidence | −0·14 (1·28) | 1·06 (1·21) | 1·53 (1·71) | 1·73 (0·73) | 3·99 | 0·263 |
| DC incidence | −0·70 (1·24) | 0·08 (1·49) | 1·53 (1·67) | 2·16 (0·92) | 7·89 | 0·048 |

Values are median EAPC (IQR) across countries and areas within each analytical subregion. Differences in EAPC distributions across subregions were assessed using Kruskal–Wallis tests. For DC incidence, the significant omnibus test was followed by Dunn’s pairwise comparisons with Holm adjustment; no pairwise comparison remained statistically significant after adjustment (all adjusted P>0·05). HCC and DC analyses included 32 countries and areas because EAPCs could not be estimated for Niue and Tokelau, for which modelled rates were zero throughout the study period. Chronic incidence and mortality analyses included all 34 countries and areas. EAPC=estimated annual percentage change. HCC=hepatocellular carcinoma. DC=decompensated cirrhosis. PICTs=Pacific Island countries and territories.

## Supplementary Table 3. Exploratory associations between hepatitis B vaccination coverage and changes in modelled HBV burden across countries and areas with available vaccination data, 2015–2024

| **Vaccination measure** | **HBV burden outcome** | **n** | **Spearman ρ** | **Unadjusted P value** | **Holm-adjusted P value** |
| --- | --- | --- | --- | --- | --- |
| HepB-BD coverage in 2015 | Under-five HBV  prevalence change | 28 | 0·26 | 0·19 | 0·94 |
| HepB3 coverage in 2015 | Under-five HBV  prevalence change | 28 | −0·18 | 0·37 | 1·00 |
| HepB-BD coverage in 2015 | Chronic HBV  incidence EAPC | 28 | 0·19 | 0·35 | 1·00 |
| HepB3 coverage in 2015 | Chronic HBV  incidence EAPC | 28 | −0·07 | 0·72 | 1·00 |
| Change in HepB-BD coverage | Under-five HBV  prevalence change | 28 | −0·30 | 0·12 | 0·84 |
| Change in HepB3 coverage | Under-five HBV  prevalence change | 28 | −0·30 | 0·12 | 0·84 |
| Change in HepB-BD coverage | Chronic HBV  incidence EAPC | 28 | −0·25 | 0·20 | 0·94 |
| Change in HepB3 coverage | Chronic HBV  incidence EAPC | 28 | −0·44 | 0·02 | 0·17 |

Associations were assessed using Spearman rank correlation. Changes in HepB-BD and HepB3 coverage were calculated as the absolute percentage-point difference between 2015 and 2024 coverage. Changes in under-five HBV prevalence were calculated as the percentage change between 2015 and 2024; negative values indicate declining prevalence. Chronic HBV incidence trends were expressed as estimated annual percentage changes (EAPCs), with negative values indicating declining incidence. Holm adjustment was applied across the eight exploratory comparisons to account for multiple testing. Vaccination data were unavailable for American Samoa, Guam, New Caledonia, Northern Mariana Islands, Tokelau, and Wallis and Futuna; these settings were excluded from this analysis. In a sensitivity analysis excluding Japan, which experienced a marked increase in HepB3 coverage during the study period, the association between change in HepB3 coverage and chronic HBV incidence remained inverse but was attenuated (ρ=−0.370, P=0.057). EAPC=estimated annual percentage change; HBV=hepatitis B virus; HepB-BD=hepatitis B birth-dose vaccination; HepB3=three-dose hepatitis B vaccination.

## Supplementary Table 4. Sensitivity analysis of country-level HBV burden trends according to World Bank FY2025 income classification, 2015–2024

| **Outcome** | **Lower-middle income (n=10), median EAPC (IQR)** | **Upper-middle income (n=7), median EAPC (IQR)** | **High income (n=13), median EAPC (IQR)** | **Kruskal–Wallis χ²** | **P value** |
| --- | --- | --- | --- | --- | --- |
| Chronic HBV incidence | −5·60 (5·06) | −6·64 (7·28) | −6·92 (3·76) | 2·97 | 0·227 |
| HBV-related mortality | 1·57 (1·57) | 2·23 (2·51) | 2·21 (1·47) | 1·03 | 0·597 |
| HCC incidence | 1·61 (1·78) | 1·53 (1·96) | 1·34 (1·14) | 0·94 | 0·625 |
| DC incidence | 1·90 (1·34) | 1·87 (2·15) | 1·02 (1·79) | 3·79 | 0·150 |

Countries and areas were classified according to the World Bank FY2025 income classification, corresponding to calendar-year 2023 GNI per capita. Four countries and areas without an FY2025 World Bank income classification (Cook Islands, Niue, Tokelau, and Wallis and Futuna) were excluded from this sensitivity analysis. Country-level EAPCs for chronic HBV incidence, HBV-related mortality, HCC incidence, and DC incidence were compared across lower-middle-, upper-middle-, and high-income groups using Kruskal–Wallis tests. No post-hoc pairwise comparisons were performed because none of the global tests indicated statistically significant differences across income groups. EAPC=estimated annual percentage change; HBV=hepatitis B virus; HCC=hepatocellular carcinoma; DC=decompensated cirrhosis; IQR=interquartile range; GNI=gross national income.

## Supplementary Table 5. Sensitivity analyses of the association between estimated annual percentage changes in chronic HBV incidence and HBV-related mortality, 2015–2024

| **Analysis** | **n** | **Pearson r** | **95% CI** | **Pearson P value** | **β** | **R²** | **Regression P value** |
| --- | --- | --- | --- | --- | --- | --- | --- |
| Primary analysis | 34 | 0·39 | 0·06 - 0·64 | 0·023 | 0·15 | 0·15 | 0·023 |
| Excluding Marshall Islands | 33 | 0·40 | 0·06 - 0·65 | 0·022 | 0·13 | 0·16 | 0·022 |
| Shared model-derived trajectories collapsed | 27 | 0·40 | 0·02 - 0·68 | 0·039 | 0·15 | 0·16 | 0·039 |
| Excluding substantially nonlinear chronic-incidence trajectories | 31 | 0·49 | 0·16 - 0·72 | 0·006 | 0·20 | 0·24 | 0·006 |

Pearson correlation and simple linear regression were used to assess the association between country-level chronic HBV incidence EAPCs and HBV-related mortality EAPCs. Three sensitivity analyses were conducted. First, the analysis was repeated after excluding the Marshall Islands, which had the highest HBV-related mortality EAPC. Second, identical model-derived chronic HBV incidence and mortality trajectories shared by several Pacific Island countries and areas were each represented by a single analytical observation, reducing the dataset from 34 countries and areas to 27 unique trajectory units. Third, the suitability of the log-linear EAPC model was assessed by comparing country-specific log-linear models with models containing an additional quadratic time term. For this sensitivity analysis, substantial nonlinearity was defined as both P<0·05 for the quadratic term and an increase in R² greater than 0·05 relative to the log-linear model. Three countries and areas met these criteria for chronic HBV incidence (Papua New Guinea, New Caledonia, and Tuvalu) and were excluded, leaving 31 observations. The direction of the association was unchanged, and effect estimates were similar or stronger across the three sensitivity analyses, indicating that the primary association was not materially dependent on the country with the highest mortality EAPC, repeated shared model-derived trajectories, or chronic incidence trajectories showing substantial departure from log-linearity. CI=confidence interval. EAPC=estimated annual percentage change. HBV=hepatitis B virus.

## Supplementary Table 6. Comparison of hepatitis B burden estimates from the study analytical dataset and the WHO Global hepatitis report 2026, Western Pacific Region

| **Domain** | **Present study: CDA Foundation WPR estimates, 2015–2024** | **WHO Global hepatitis report 2026** | **Shen et al. (2026), analysis of GBD 2021 estimates** | **Interpretation** |
| --- | --- | --- | --- | --- |
| Chronic HBV burden / prevalence | Total HBV prevalence declined from 5·28% (95% UI 4·23–6·34) in 2015 to 4·58% (3·66–5·49) in 2024; 89·7 million people were estimated to be living with HBV in 2024 | 102 million (95% UI 92–117) people were estimated to be living with chronic HBV in the WPR in 2024 | Globally, prevalent CHB cases declined 8·32% during 1990–2021, with a significant decline in age-standardised prevalence; East Asia showed among the largest reductions | All sources indicate a substantial continuing HBV burden despite long-term reductions. Absolute estimates from different modelling rounds/frameworks are not directly interchangeable |
| Chronic HBV incidence | Chronic HBV incidence declined from 6·32 to 3·16 per 100 000 during 2015–2024 | The report describes continuing reductions in new HBV infections but does not provide a directly comparable WPR chronic-incidence estimate for the present analytical definition | Globally, CHB incident cases declined 38·45% during 1990–2021 and age-standardised incidence declined significantly (AAPC −2·61%) | Directionally consistent evidence of declining incidence, although outcome definitions, periods, and geographical groupings differ |
| HBV-related mortality | HBV-related deaths increased from 436 363 to 484 055; regional mortality rate increased from 22·88 to 24·70 per 100 000 during 2015–2024 | 527 000 (95% UI 476 000–593 000) HBV-related deaths in the WPR in 2024; mortality rate increased from 22 to 24 per 100 000 during 2015–2024 (+7·9%) | Globally, CHB-related deaths increased 20·04% during 1990–2021, despite a significant decline in age-standardised mortality (AAPC −1·83%) | WHO shows close agreement with the direction and magnitude of the regional mortality-rate trend. Independent GBD estimates similarly demonstrate that increasing absolute mortality can coexist with declining age-standardised mortality |
| Under-five HBV burden | Under-five prevalence declined but plateaued at approximately 0·28–0·29% from 2019, remaining above the WHO 2030 target of ≤0·1% | WPR under-five prevalence is shown at approximately 0·3% in 2024 and remains above the 2030 target* | GBD analyses indicate substantial long-term reductions in HBV burden but continued regional heterogeneity; Oceania and Southeast Asia were projected to remain off-track for some elimination indicators | Consistent evidence of substantial prevention gains alongside residual childhood HBV burden and uneven progress towards elimination |
| Ageing and interpretation of mortality | Age-standardised mortality was unavailable; ageing and longstanding chronic infection were therefore considered possible contributors rather than tested explanations | WHO identifies continuing HBV mortality as a major elimination challenge despite reductions in new infections | GBD found increasing absolute deaths despite declining age-standardised mortality, with mortality burden increasingly concentrated at older ages | Independent age-standardised evidence supports demographic change as an important consideration when interpreting mortality trends, but does not establish the mechanism underlying the present study’s findings |
| Geographical heterogeneity | Country-level trajectories were heterogeneous; analytical subregion and income classification explained little of the observed variation in EAPCs | WHO reports substantial variation in burden and progress within the WPR | East Asia and Southeast Asia remained high-burden GBD regions despite long-term improvements; Oceania showed substantial remaining gaps in reducing incident infections | Findings across sources support the importance of looking beyond regional averages, although WHO, GBD, and study geographical classifications are not directly equivalent |
| Progress towards elimination | Prevention gains were substantial, but incidence, mortality, childhood prevalence, and country-level trajectories showed different patterns of progress | WHO concludes that progress remains insufficient to achieve the 2030 viral hepatitis elimination goals | Most GBD regions were projected not to achieve all WHO 2030 CHB elimination targets | Broadly consistent evidence that progress is heterogeneous across elimination dimensions and that improvements in transmission cannot be assumed to correspond to contemporaneous reductions in mortality |

Data sources and estimates are not directly interchangeable. The present study used CDA Foundation modelled estimates for 34 countries and areas in the WHO Western Pacific Region (WPR) during 2015–2024. The WHO Global hepatitis report 2026 presents updated model-based estimates, including estimates produced with support from the CDA Foundation; comparison with WHO estimates therefore assesses consistency with the subsequent WHO estimation round rather than independent external validation (1). Shen et al. (2026) analysed independent Global Burden of Disease (GBD) 2021 estimates for 1990–2021 using GBD regional classifications, age-standardised rates, and Joinpoint regression (15). GBD regions do not correspond directly to the WHO WPR or the analytical subregions used in the present study. Comparisons are therefore descriptive and intended to assess consistency in the direction and broader epidemiological pattern of findings rather than numerical agreement between estimates. The WHO WPR under-five prevalence estimate is presented graphically in the Global hepatitis report 2026 and is therefore shown approximately. AAPC=average annual percentage change; CDA=Center for Disease Analysis Foundation; CHB=chronic hepatitis B; GBD=Global Burden of Disease; HBV= hepatitis B virus.
